# Physical activity pattern in Iran: Findings from STEPS 2021

**DOI:** 10.3389/fpubh.2022.1036219

**Published:** 2023-01-04

**Authors:** Seyed Aria Nejadghaderi, Naser Ahmadi, Mohammad-Mahdi Rashidi, Ali Ghanbari, Maryam Noori, Mohsen Abbasi-Kangevari, Maryam Nasserinejad, Negar Rezaei, Moein Yoosefi, Nima Fattahi, Erfan Ghasemi, Yosef Farzi, Elham Abdolhamidi, Mahbobeh Darman, Shirin Djalalinia, Farshad Farzadfar

**Affiliations:** ^1^Non-communicable Diseases Research Center, Endocrinology and Metabolism Population Sciences Institute, Tehran University of Medical Sciences, Tehran, Iran; ^2^Systematic Review and Meta-Analysis Expert Group (SRMEG), Universal Scientific Education and Research Network (USERN), Tehran, Iran; ^3^Student Research Committee, School of Medicine, Iran University of Medical Sciences, Tehran, Iran; ^4^Center for Life Course Heath Research, Faculty of Medicine, University of Oulu, Oulu, Finland; ^5^Endocrinology and Metabolism Research Center, Endocrinology and Metabolism Clinical Sciences Institute, Tehran University of Medical Sciences, Tehran, Iran; ^6^Department of Internal Medicine, Yale School of Medicine, New Haven, CT, United States; ^7^Deputy of Health, Ministry of Health and Medical Education, Tehran, Iran; ^8^Deputy of Research and Technology, Ministry of Health and Medical Education, Tehran, Iran

**Keywords:** physical activity, STEPwise approach to risk factor surveillance, sedentary behavior, Iran, cross sectional analysis

## Abstract

**Background:**

Insufficient physical activity (IPA) is a significant risk factor for various non-communicable diseases. The Iran action plan is a 20% reduction in IPA. Therefore, we aimed to describe the age and sex pattern of physical activity domains, IPA, the intensity of physical activity, sedentary behavior, and their associates at Iran's national and provincial levels in 2021.

**Methods:**

This study used the data of the STEPwise Approach to NCD Risk Factor Surveillance (STEPS) 2021 in Iran. The STEPS study used the Global Physical Activity Questionnaire (GPAQ) version two developed by WHO for the assessment of physical activity, which included work, transport, and recreational activities domains. We showed and compared demographic and clinical characteristics of participants between males and females, using *t*-test and Chi-square test. A logistic regression model adjusted for residential areas, years of schooling, wealth index, age, marital status, and occupation has also been implemented. The results were presented as percentages and 95% confidence intervals (CI).

**Results:**

We included 27,874 participants with a mean (SD) age of 45.69 (15.91), among whom 12,479 (44.77%) were male. The mean prevalence of IPA for the whole population for all ages was 51.3% (50.62–51.98%). By sex, 41.93% (40.88–42.98%) and 57.87% (56.99–58.75%) of men and women had IPA, respectively. According to the physical activity domains, the age-standardized prevalence of no recreational activity was 79.40% (78.80–79.99%), no activity at work was 66.66% (65.99–67.32%), and no activity at transport was 49.40% (48.68–50.11%) for both sexes combined. Also, the overall age-standardized prevalence of sedentary behaviors was 50.82% (50.11–51.53%). Yazd province represented the highest prevalence of IPA (63.45%), while West Azerbaijan province represented the lowest prevalence (39.53%). Among both sexes, living in the urban area vs. rural area [adjusted OR: 1.44; (1.31–1.58)], married vs. single status [adjusted OR: 1.33; (1.16–1.53)], and wealth index of class 3 vs. class 1 [adjusted OR: 1.15; (1.01–1.30)] were significantly associated with a higher rate of IPA.

**Conclusion:**

The prevalence of IPA was considerably high in Iran. To achieve the predefined goal of reducing IPA, the health system should prioritize increasing physical activity, especially in urban areas and among females.

## Introduction

Insufficient physical activity (IPA) is a significant public health issue associated with a wide range of non-communicable diseases like cancers, diabetes, ischemic stroke, and ischemic heart disease (1). Moreover, a higher level of physical activity is associated with lower occurrence and mortality of infectious diseases (2). According to the Global Burden of Disease (GBD) study estimates, the global prevalence of low physical activity has increased by 0.2% from 1990 to 2019 (3).

In Iran, 0.46% of total health-related costs were directly attributable to IPA in 2013 (4). Furthermore, IPA was responsible for 4.4% and 1.9% of deaths and disability-adjusted life-years (DALYs) attributable to non-communicable diseases (NCDs) in 2019 in Iran, respectively (5). Also, a 20% relative reduction in IPA by 2025 is one of the action plans for controlling NCDs developed by the Iranian Non-Communicable Diseases Committee (INCDC) (5). Furthermore, regarding sustainable development goals (SDG) 3.4, a reduction by one-third in premature mortality from NCDs through prevention and treatment and promoting mental health and wellbeing was targeted to reach by 2030 (6). In line, the global action plan was set to a 15% relative reduction in the global prevalence of physical inactivity in adults and in adolescents by 2030 (7).

Based on the World Health Organization's (WHO) STEPwise approach to risk factor Surveillance (STEPS), Iran has established a national survey program since 2005 to provide reliable and up-to-date information for health policymakers (8). Previously, the physical activity profile of the Iranian adult population has been reported using STEPS 2016 (9). Moreover, another previously published study reported the prevalence of low physical activity in Iran, using data from a national survey in 2011 (10). The prevalence of low physical activity in Iran has been reported using a systematic review study in 2016 (11), and it was reported among Iranian adolescents between 2006 and 2011 (12). Also, another study reported the burden of diseases, injuries and risk factors in Iran using data of the Global Burden of Disease 2019 project (13). Nevertheless, the studies need to be updated to provide the most up-to-date data for health policymakers. Herein, we aimed to update the patterns of physical activity domains, IPA, the intensity of physical activity, and sedentary behavior at the national and provincial level of Iran in 2021. Also, the associates of physical activity in Iran were determined.

## Methods

### Overview

This study used the data of the STEPwise Approach to NCD Risk Factor Surveillance (STEPS) 2021, which was based on the platform of the WHO STEPwise approach to NCD risk factor surveillance (14). This program aims to collect comprehensive data and analyze and interpret them to provide reliable information on the prevalence of significant risk factors for health policymakers. Following seven rounds of STEPS in Iran in 2005, 2006, 2007, 2008, 2009, 2011, and 2016, the recently updated round was conducted in 2021. Further details on the study methodology has been previously published (15).

### Study design and participants

The present study is cross-sectional and was initiated in early 2020. However, it was temporarily suspended due to the coronavirus disease 2019 (COVID-19) pandemic, when just about 10% of data were collected. Other remaining 90% of samples were collected during the COVID-19 pandemic. Moreover, the completion of sampling was continued after the third peak of COVID-19 when the incidence rate of COVID-19 reached its lowest levels. The 10% of participants who were enrolled before the initiation of COVID-19 pandemic were also included in the analysis.

In order to include the usual sample size, 3,176 clusters based on provinces' population and relative weighting were calculated. We calculated a sample size of 28,821 from both rural and urban areas. Initially, it was calculated to include 10 participants in each cluster, but due to the COVID-19 limitations, each cluster was completed with 9 participants. All Iranian adults aged ≥18 were included as the target population. People with psychological problems who may be unable to answer the questionnaire, people for whom anthropometry measurement was impossible due to physical problems, people who could not provide laboratory samples, and pregnant women were excluded from the study. Data on demographic factors and metabolic and behavioral risk factors were collected from the included participants.

### Variables

The study was conducted in three consecutive steps. In the first step, data on demographic features, diet, physical activity, past medical history, habitual history, health-related quality of life, cancer screening, and family history of participants were collected using a questionnaire. In the second step, physical measurements were gathered, including height, weight, waist and hip circumference, blood pressure, and pulse rate. Lastly, laboratory measures like total serum cholesterol, serum high-density lipoprotein cholesterol (HDL-C), serum triglyceride, and fasting plasma glucose (FPG), as well as anti-SARS-CoV-2 IgG test, were evaluated in the third step.

### Definitions

We used the Global Physical Activity Questionnaire (GPAQ) version two, developed by WHO, for the assessment of physical activity and sedentary behaviors (16). Based on GPAQ, physical activity is categorized into work-, transport-, and recreational-domains which was assessed through direct interviews with eligible participants according to the STEPS protocol (14). The validity and reliability of GPAQ have been evaluated in the previous publications, representing generally acceptable strength (17, 18).

In order to measure the domains of physical activity, the metabolic equivalent of task (MET) scores were calculated according to the GPAQ Instrument and Analysis Guide v2 (16). One MET is equivalent to one kilocalorie per kilogram per hour energy consumption when the body is at rest, reviving 3.5 ml/kg/min of oxygen (19). The GPAQ categorized activities into moderate- and vigorous-intensity activities based on the assignment of the MET scores equivalent to 4 and 8, respectively. Above 600 METs per week was defined as sufficient physical activity, which was 150 or 75 min of moderate- and vigorous-intensity physical activity per week, respectively, or a combination of both activities. IPA was defined as a combination of physical activities of <600 METs per week (20). Total work-, transport-, and recreational-related activities were defined as overall related physical activity in minutes per week. Total physical activity was defined as the sum of the total MET minutes of all physical activity domains. Sedentary lifestyle was considered if the participant reported more than 4 h per day of sedentary situations like sitting (21).

Moreover, education was defined as the number of successfully completed years of schooling and categorized into four subgroups [i.e., 0 (Illiterate), 1–6, 7–11, and 12 years and over]. We used the principal component analysis method to calculate the wealth index by using the data related to home area, the number of rooms in the house, family assets, and home appliances that the family possessed in the questionnaire. The calculated wealth indices of participants were categorized into five quintiles, from the poorest (first quintile) to the richest (fifth quintile). Diabetes based on FPG was defined as FPG ≥ 126 mg/dl or self-reported based on the intake of oral hypoglycemic agents and/or insulin injection (22). Hypertension was defined as a systolic blood pressure ≥140 mmHg, or diastolic blood pressure ≥90 mmHg, or self-reported drug intake (23). Dyslipidemia was defined as cholesterol >240 mg/dl, triglyceride >150 mg/dl, or HDL-C <40 mg/dl for males or <50 mg/dl for females, low-density lipoprotein cholesterol (LDL-C) >100, or using an oral agent for hypercholesterolemia (24). Data on cardiovascular diseases were based on self-reports.

### Statistical analysis

Data cleaning was performed in three steps (25). Regarding the handling of missing data, the whole section was regarded as missing data in the event that the response to the primary question in each part of the questionnaire—typically the first question in each section—was not provided. Both data cleaning and handling of missing data processes were conducted by two biostatisticians, and any discrepancies were resolved by consultation with a third data expert. More details have been published elsewhere (15).

We used descriptive statistics to report the prevalence of each physical activity domain and IPA. In addition, we showed and compared demographic and clinical characteristics of participants between males and females, using *t*-test and Chi-square test. Also, different logistic regression models were used to assess the roles of different demographic factors and comorbidities as independent variables on IPA as dependent variable in Iran. The models were adjusted for the effects of socioeconomic factors (i.e., residential areas, years of schooling, wealth index, age, marital status, and occupation). We used a stepwise approach to determine the relevant covariates in which variables with *p* < 0.05 and missing data <30% were included. Also, the random effect regression model was used. Quantitative and qualitative variables were shown as the mean ± standard deviation (SD) and number (percentage), respectively. Since there were different total number for each comorbidity or other demographic factors, the numbers and percentages are different Statistical analyses were performed using Stata version 14 (Stata Corporation, College Station, TX, USA) and R software (R Foundation for Statistical Computing, Vienna, Austria). *P*-values < 0.05 were considered statistically significant. The names of Iran's provinces on the map were provided in Supplementary Presentation 1.

### Ethical considerations

Ethical approval for the study was obtained from the ethics committee of Tehran University of Medical Sciences, Tehran, Iran (IR.TUMS.NIHR.REC.1398.006). Participation in the study was voluntary. The study's aims and methods were described for all eligible individuals and written informed consent was obtained from all those who agreed to participate. The funding source had no role in the study design, data collection, analysis, data interpretation, manuscript writing, and the decision on paper submission.

## Results

### Baseline characteristics

**Figure 1:**
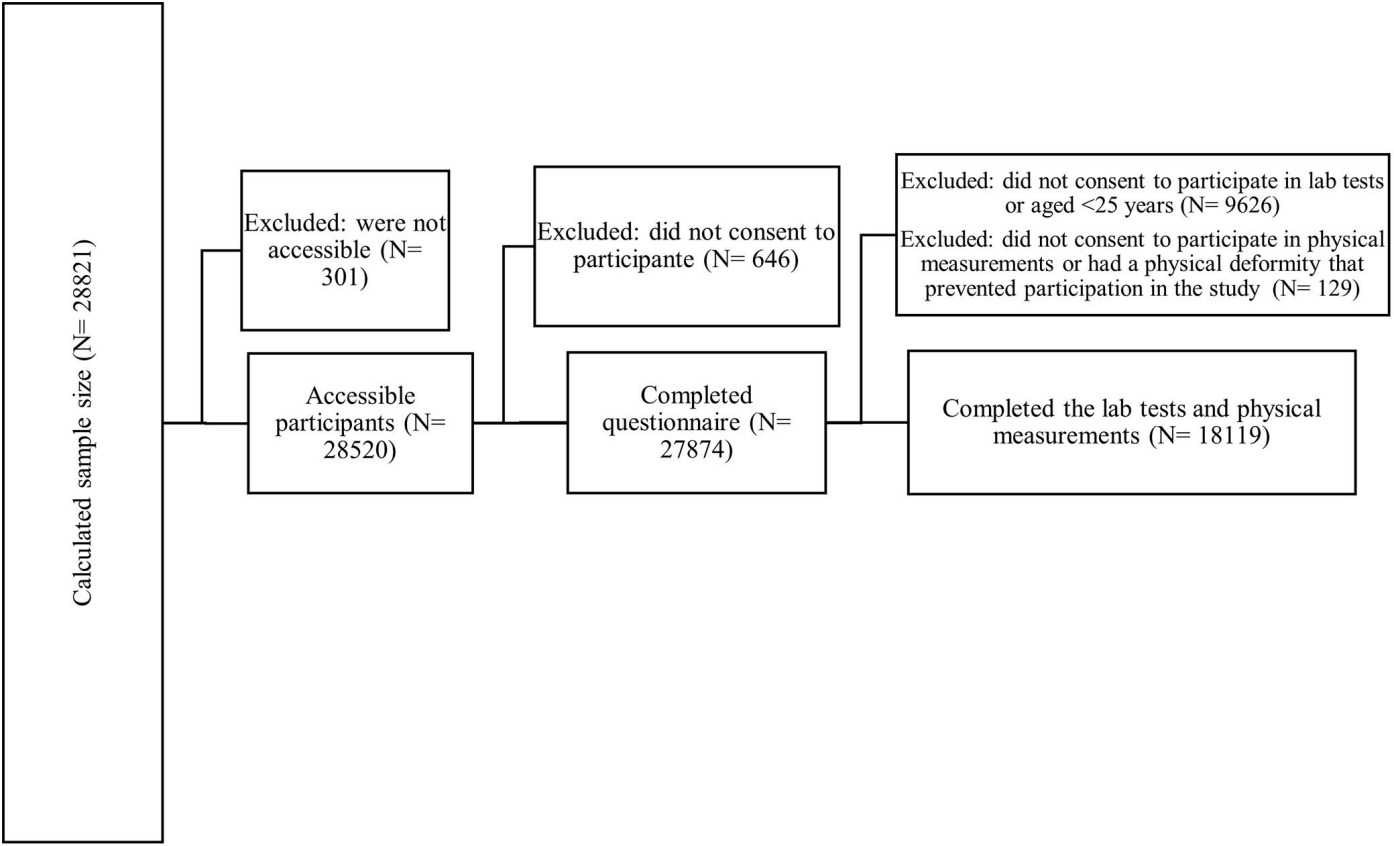
Flow diagram of study selection.

We calculated a sample size of 28,821 participants, and after the exclusion of 947 participants (301 who were not accessible and 646 who did not consent for participation), finally 27,874 participants were completed the questionnaire and were included (Figure 1). The mean age was 45.69 (15.91), and 12,479 (44.77%) were male. Most participants (74.97%) lived in urban areas. Also, 21,359 (76.59%) were married, 11,722 (43.65%) had 12 or over years of schooling, and 13,496 (48.47%) had unpaid jobs. Furthermore, 24,969 (89.63%) had basic health insurance, 7,608 (28.42%) had complementary insurance, and 5,265 (21.4%) were of class 5 wealth index (Table 1). The prevalence of overweight, hypertension, dyslipidemia, diabetes, and cardiovascular disease among participants were 10,485 (38.06%), 18,717 (67.97%), 12,865 (71.21%), 2,443 (14.15%), and 1,840 (6.71%), respectively. There was a significant difference in prevalence of hypertension (*p* = 0.001), cardiovascular diseases (*p* < 0.001), dyslipidemia (*p* < 0.001), and different BMI categories (*p* < 0.001) between males and females, while the prevalence of diabetes did not have a significant difference between sexes (*p* = 0.095) (Table 2).

**Table 1:** Baseline characteristics of participants.

**Variables**	**Female**	**Male**	**Total**	***p*** **-value**
Age, mean (SD)	45.29 (15.43)	46.19 (16.48)	45.69 (15.91)	< 0.001
**Residential area**, ***n*** **(%)**				
Rural	4,196 (27.26%)	3,402 (27.26%)	7,598 (25.03%)	0.523
Urban	11,199 (72.74%)	9,077 (72.74%)	20,276 (74.97%)	
**Years of schooling**, ***n*** **(%)**				
Zero	2,825 (18.49%)	1,190 (9.61%)	4,015 (13.49%)	< 0.001
1–6	4,048 (26.49%)	2,695 (21.76%)	6,743 (23.78%)	
7–11	2,490 (16.29%)	2,698 (21.78%)	5,188 (19.08%)	
12 and over	5,918 (38.73%)	5,804 (46.86%)	11,722 (43.65%)	
**Marital status**, ***n*** **(%)**				
Divorced/separate with partner	414 (2.69%)	152 (1.22%)	566 (2.19%)	< 0.001
Married	11,445 (74.34%)	9,914 (79.45%)	21,359 (76.59%)	
Single	1,961 (12.74%)	2,286 (18.32%)	4,247 (15.05%)	
Widow	1,575 (10.23%)	127 (1.02%)	1,702 (6.17%)	
**Employment status**				
Freelance job or self-employed	631 (4.13%)	5,705 (46.06%)	6,336 (23.31%)	< 0.001
Private sector employee	358 (2.34%)	407 (3.29%)	765 (3.21%)	
Private sector labor	144 (0.94%)	934 (7.54%)	1,078 (3.77%)	
Public sector employee	583 (3.82%)	1,070 (8.64%)	1,653 (5.78%)	
Public sector labor	44 (0.29%)	210 (1.7%)	254 (0.88%)	
Retired	381 (2.49%)	2,157 (17.41%)	2,538 (9.31%)	
Unemployed due to disability	48 (0.31%)	398 (3.21%)	446 (1.42%)	
Unemployed not seeking work	117 (0.77%)	201 (1.62%)	318 (1.18%)	
Unemployed seeker job	222 (1.45%)	562 (4.54%)	784 (2.67%)	
Unpaid work	12,753 (83.46%)	743 (6%)	13,496 (48.47%)	
**Basic health insurance**, ***n*** **(%)**				
No	1,278 (8.36%)	1,421 (11.47%)	2,699 (10.37%)	< 0.001
Yes	14,003 (91.64%)	10,966 (88.53%)	24,969 (89.63%)	
**Complementary insurance**, ***n*** **(%)**				
No	10,960 (72%)	8,975 (72.84%)	19,935 (71.58%)	0.05
Yes	4,262 (28%)	3,346 (27.16%)	7,608 (28.42%)	
**Wealth index**				
Poorest	3,024 (21.4%)	2,244 (18.39%)	5,268 (19.04%)	< 0.001
Class 2	2,985 (21.13%)	2,282 (18.7%)	5,267 (20.64%)	
Class 3	2,707 (19.16%)	2,559 (20.97%)	5,266 (18.95%)	
Class 4	2,698 (19.1%)	2,569 (21.05%)	5,267 (19.98%)	
Richest	2,715 (19.22%)	2,550 (20.89%)	5,265 (21.4%)	

**Table 2:** Prevalence of underlying conditions among participants.

**Variable**	**Female** ***N*** **(%)**	**Male** ***N*** **(%)**	**Total** ***N*** **(%)**	***p*** **-value**
**BMI**				
< 18.5	500 (52.53%)	469 (47.47%)	969 (3.37%)	< 0.001
18.5–24.9	4,451 (47.36%)	4,974 (52.64%)	9,425 (33.61%)	
25–29.9	5,641 (53.56%)	4,844 (46.44%)	10,485 (38.06%)	
30–34.9	3,286 (65.81%)	1,702 (34.19%)	4,988 (18.19%)	
35–39.9	1,086 (76.93%)	338 (23.07%)	1,424 (5.18%)	
40+	352 (83.15%)	70 (16.85%)	422 (1.59%)	
**Hypertension (blood pressure above 140/90)**				
No	5,135 (56.91%)	3,896 (43.09%)	9,031 (32.03%)	0.001
Yes	10,207 (54.63%)	8,510 (45.37%)	18,717 (67.97%)	
**Diabetes (Ever)**				
No	8,829 (55.3%)	6,832 (44.7%)	15,661 (85.85%)	0.095
Yes	1,456 (57.86%)	987 (42.14%)	2,443 (14.15%)	
**Cardiovascular diseases (Ever)**				
No	14,530 (56.11%)	11,452 (43.89%)	25,982 (93.29%)	< 0.001
Yes	839 (45.1%)	1,001 (54.9%)	1,840 (6.71%)	
**Dyslipidemia**				
No	2,549 (47.94%)	2,679 (52.06%)	5,228 (28.79%)	< 0.001
Yes	7,731 (58.76%)	5,134 (41.24%)	12,865 (71.21%)	

### Prevalence of national and sub-national insufficient physical activity

The mean prevalence of IPA for the whole population for all ages was 51.3% (95% CI: 50.62–51.98%). By sex, 41.93% (95% CI: 40.88–42.98%) and 57.87% (95% CI: 56.99–58.75%) of males and females in all ages had IPA, respectively. Individuals aged above 75 years old had the highest prevalence of IPA [68.07% (95% CI: 64.90–71.09%)], while those in 18–24 age group had the lowest prevalence [45.5% (95% CI: 43.3–47.73%)]. In all age groups, the prevalence of IPA was higher for females. The highest difference was evident in 70–74 age group and the lowest difference was for 35–44 age group (Table 3).

**Table 3:** Prevalence of physical activity domains, sedentary behaviors, and insufficient physical activity among women and men of various age-groups in Iran.

**Age groups**	**Sex**	**Insufficient physical activity**		**No activity at work**		**No activity at transport**		**No recreational activity**		**Sedentary behaviors**		**Percentage contribution of vigorous PA in total MET**	
		***N*** **(%)**	**95% CI**	***N*** **(%)**	**95% CI**	***N*** **(%)**	**95% CI**	***N*** **(%)**	**95% CI**	***N*** **(%)**	**95% CI**	***N*** **(%)**	**95% CI**
All ages	Female	8,531 (57.87%)	(56.99, 58.75)%	11,358 (74.42%)	(73.65, 75.17)%	8,048 (52.94%)	(52.06, 53.82)%	13,570 (88.19%)	(87.6, 88.75)%	7,696 (50.43%)	(49.55, 51.31)%	195 (1.32%)	(1.13, 1.54)%
	Male	4,278 (41.93%)	(40.88, 42.98)%	7,148 (58.44%)	(57.48, 59.4)%	5,627 (45.35%)	(44.38, 46.33)%	9,270 (74.98%)	(74.13, 75.82)%	6,200 (49.4%)	(48.43, 50.38)%	923 (7.06%)	(6.58, 7.57)%
	Total	12,809 (51.3%)	(50.62, 51.98)%	18,506 (67.28%)	(66.68, 67.89)%	13,675 (49.55%)	(48.9, 50.21)%	22,840 (82.29%)	(81.78, 82.78)%	13,896 (49.97%)	(49.32, 50.63)%	1,118 (3.88%)	(3.64, 4.14)%
	*p*-value	< 0.001		< 0.001		< 0.001		< 0.001		0.126		< 0.001	
Age-standardized	Female	8,531 (57.1%)	(56.12, 58.07)%	11,358 (74.48%)	(73.64, 75.29)%	8,048 (52.3%)	(51.34, 53.27)%	13,570 (86.35%)	(85.63, 87.04)%	7,696 (51.95%)	(50.99, 52.9)%	195 (1.57%)	(1.33, 1.85)%
	Male	4,278 (40.57%)	(39.42, 41.73)%	7,148 (56.97%)	(55.91, 58.02)%	5,627 (45.79%)	(44.72, 46.87)%	9,270 (70.78%)	(69.77, 71.77)%	6,200 (49.42%)	(48.34, 50.49)%	923 (8.63%)	(8.01, 9.29)%
	Total	12,809 (50.35%)	(49.6, 51.11)%	18,506 (66.66%)	(65.99, 67.32)%	13,675 (49.4%)	(48.68, 50.11)%	22,840 (79.4%)	(78.8, 79.99)%	13,896 (50.82%)	(50.11, 51.53)%	1,118 (4.72%)	(4.41, 5.06)%
	*p*-value	< 0.001		< 0.001		< 0.001		< 0.001		0.001		< 0.001	
18–24 years	Female	771 (56.83%)	(53.87, 59.75)%	1,077 (76.93%)	(74.4, 79.27)%	678 (48.36%)	(45.45, 51.28)%	1,091 (77.87%)	(75.34, 80.21)%	939 (67.13%)	(64.35, 69.8)%	36 (2.89%)	(2.04, 4.07)%
	Male	314 (30.83%)	(27.8, 34.04)%	733 (58.01%)	(54.94, 61.02)%	513 (40.59%)	(37.6, 43.66)%	607 (49.4%)	(46.34, 52.46)%	685 (53.91%)	(50.82, 56.97)%	187 (14.43%)	(12.39, 16.73)%
	Total	1,085 (45.5%)	(43.3, 47.73)%	1,810 (67.98%)	(65.99, 69.91)%	1,191 (44.69%)	(42.59, 46.8)%	1,698 (64.4%)	(62.36, 66.39)%	1,624 (60.88%)	(58.8, 62.92)%	223 (8.35%)	(7.24, 9.6)%
	*p*-value	< 0.001		< 0.001		< 0.001		< 0.001		< 0.001		< 0.001	
25–34 years	Female	1,596 (57.3%)	(55.25, 59.32)%	2,160 (74.67%)	(72.88, 76.39)%	1,530 (53.67%)	(51.64, 55.69)%	2,435 (84.14%)	(82.6, 85.57)%	1,554 (53.64%)	(51.61, 55.66)%	53 (1.93%)	(1.44, 2.59)%
	Male	681 (38.15%)	(35.7, 40.66)%	1,175 (51.56%)	(49.31, 53.81)%	1,095 (47.67%)	(45.41, 49.94)%	1,489 (64.4%)	(62.2, 66.54)%	1,145 (49.26%)	(46.99, 51.53)%	292 (11.78%)	(10.42, 13.29)%
	Total	2,277 (49.73%)	(48.13, 51.33)%	3,335 (64.42%)	(62.98, 65.84)%	2,625 (51.01%)	(49.5, 52.52)%	3,924 (75.38%)	(74.06, 76.66)%	2,699 (51.7%)	(50.19, 53.21)%	345 (6.3%)	(5.62, 7.06)%
	*p*-value	< 0.001		< 0.001		< 0.001		< 0.001		0.005		< 0.001	
35–44 years	Female	1,837 (53.99%)	(52.13, 55.83)%	2,552 (71.92%)	(70.27, 73.51)%	1,781 (50.69%)	(48.87, 52.51)%	3,111 (86.79%)	(85.48, 87.99)%	1,571 (44.26%)	(42.45, 46.08)%	42 (1.28%)	(0.93, 1.77)%
	Male	845 (42.04%)	(39.7, 44.42)%	1,341 (52.71%)	(50.58, 54.83)%	1,284 (49.81%)	(47.67, 51.94)%	1,917 (74.28%)	(72.37, 76.1)%	1,195 (44.64%)	(42.53, 46.76)%	220 (7.97%)	(6.9, 9.2)%
	Total	2,682 (49.47%)	(48.01, 50.94)%	3,893 (63.83%)	(62.5, 65.14)%	3,065 (50.32%)	(48.94, 51.7)%	5,028 (81.52%)	(80.42, 82.57)%	2,766 (44.42%)	(43.05, 45.8)%	262 (4.1%)	(3.59, 4.68)%
	*p*-value	< 0.001		< 0.001		0.537		< 0.001		0.791		< 0.001	
45–54 years	Female	1,583 (54.57%)	(52.58, 56.54)%	2,185 (71.71%)	(69.92, 73.42)%	1,464 (48.24%)	(46.28, 50.21)%	2,800 (90.2%)	(88.95, 91.32)%	1,390 (46.19%)	(44.24, 48.15)%	39 (1.14%)	(0.79, 1.62)%
	Male	776 (42.19%)	(39.75, 44.67)%	1,223 (54.39%)	(52.14, 56.62)%	1,031 (45.72%)	(43.47, 47.99)%	1,823 (79.49%)	(77.6, 81.26)%	1,077 (46.14%)	(43.89, 48.4)%	136 (5.75%)	(4.78, 6.91)%
	Total	2,359 (49.77%)	(48.22, 51.32)%	3,408 (64.34%)	(62.92, 65.73)%	2,495 (47.17%)	(45.69, 48.65)%	4,623 (85.64%)	(84.57, 86.66)%	2,467 (46.17%)	(44.69, 47.65)%	175 (3.1%)	(2.63, 3.66)%
	*p*-value	< 0.001		< 0.001		0.1		< 0.001		0.973		< 0.001	
55–64 years	Female	1,386 (58.28%)	(56.09, 60.44)%	1,803 (73.6%)	(71.64, 75.46)%	1,318 (53.63%)	(51.45, 55.8)%	2,273 (91.84%)	(90.53, 92.98)%	1,156 (47%)	(44.81, 49.19)%	18 (0.74%)	(0.44, 1.23)%
	Male	775 (43.63%)	(41.12, 46.17)%	1,263 (63.08%)	(60.76, 65.35)%	851 (41.2%)	(38.85, 43.58)%	1,700 (82.93%)	(81.05, 84.66)%	1,038 (50.28%)	(47.88, 52.68)%	69 (3.45%)	(2.66, 4.45)%
	Total	2,161 (51.98%)	(50.32, 53.64)%	3,066 (68.83%)	(67.34, 70.28)%	2,169 (47.99%)	(46.38, 49.6)%	3,973 (87.8%)	(86.69, 88.82)%	2,194 (48.49%)	(46.87, 50.1)%	87 (1.97%)	(1.56, 2.47)%
	*p*-value	< 0.001		< 0.001		< 0.001		< 0.001		0.048		< 0.001	
65–69 years	Female	598 (65.89%)	(62.45, 69.18)%	727 (79.02%)	(76.06, 81.7)%	568 (61.04%)	(57.53, 64.43)%	887 (94.83%)	(92.95, 96.23)%	481 (52.44%)	(48.9, 55.95)%	4 (0.55%)	(0.2, 1.5)%
	Male	308 (44.67%)	(40.54, 48.87)%	523 (69.47%)	(65.76, 72.95)%	321 (40.54%)	(36.68, 44.52)%	678 (89.23%)	(86.57, 91.41)%	418 (56.46%)	(52.51, 60.33)%	11 (1.11%)	(0.56, 2.2)%
	Total	906 (56.82%)	(54.11, 59.49)%	1,250 (74.78%)	(72.48, 76.94)%	889 (51.93%)	(49.27, 54.58)%	1,565 (92.34%)	(90.82, 93.63)%	899 (54.23%)	(51.61, 56.83)%	15 (0.8%)	(0.45, 1.42)%
	*p*-value	< 0.001		< 0.001		< 0.001		< 0.001		0.138		0.248	
70–74 years	Female	374 (72.81%)	(68.26, 76.92)%	431 (84.27%)	(80.49, 87.43)%	342 (65.64%)	(60.89, 70.09)%	497 (96.11%)	(93.55, 97.68)%	302 (59.59%)	(54.79, 64.21)%	2 (0.43%)	(0.1, 1.89)%
	Male	230 (46.75%)	(41.88, 51.69)%	381 (73.55%)	(69.2, 77.49)%	230 (43.7%)	(39.02, 48.49)%	478 (91.17%)	(87.89, 93.62)%	291 (55.48%)	(50.7, 60.16)%	3 (0.62%)	(0.18, 2.13)%
	Total	604 (59.89%)	(56.46, 63.22)%	812 (78.83%)	(75.98, 81.42)%	572 (54.5%)	(51.1, 57.85)%	975 (93.6%)	(91.63, 95.14)%	593 (57.51%)	(54.14, 60.81)%	5 (0.53%)	(0.2, 1.36)%
	*p*-value	< 0.001		< 0.001		< 0.001		0.006		0.23		0.723	
75+ years	Female	386 (79.71%)	(75.41, 83.43)%	423 (87.04%)	(83.32, 90.03)%	367 (76.08%)	(71.59, 80.06)%	476 (98.11%)	(95.97, 99.13)%	303 (62.16%)	(57.21, 66.86)%	1 (0.3%)	(0.04, 2.12)%
	Male	349 (58.68%)	(54.17, 63.04)%	509 (81.8%)	(78.17, 84.94)%	302 (47.35%)	(43, 51.75)%	578 (92.4%)	(89.62, 94.48)%	351 (56.54%)	(52.19, 60.8)%	5 (1.16%)	(0.45, 2.95)%
	Total	735 (68.07%)	(64.9, 71.09)%	932 (84.08%)	(81.55, 86.32)%	669 (59.85%)	(56.58, 63.04)%	1,054 (94.88%)	(93.17, 96.19)%	654 (58.99%)	(55.75, 62.16)%	6 (0.79%)	(0.34, 1.83)%
	*p*-value	< 0.001		0.035		< 0.001		< 0.001		0.092		0.189	
	*p*-value between groups	< 0.001		< 0.001		< 0.001		< 0.001		< 0.001		< 0.001	

P-values < 0.05 represent the statistical significance.

People who lived in urban areas had a significantly higher level of IPA than those in rural areas (53.28 vs. 44.93%; *p* < 0.001). Considering the educational status, the highest IPA was in illiterate participants, whereas it was lowest among the population with 12 or higher years of schooling (59.69 vs. 48.28%; *p* < 0.001). Moreover, the prevalence of IPA was 52.02% for the wealth index of the poorest category and 49.01% for the richest category. Meanwhile, there was a significant difference in IPA by the overall wealth index (*p* = 0.015) (Supplementary Table 1).

**Figure 2:**
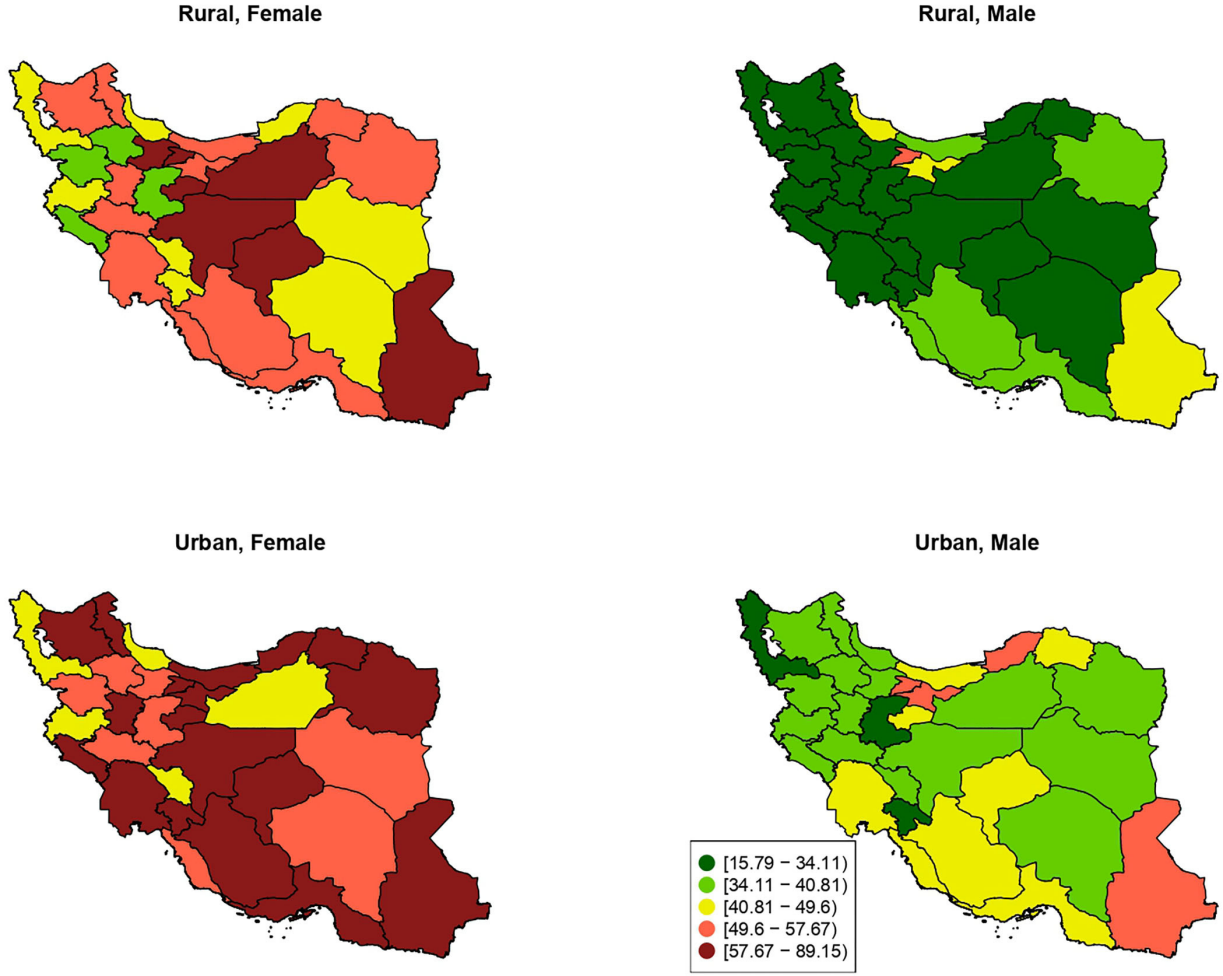
The provincial distribution of age-standardized prevalence of insufficient physical activity (percentage %) by residential area and sex.

Evaluating the IPA rate at the subnational level, Yazd, Alborz, and Sistan and Baluchistan represented the highest prevalence (63.45, 62.54, and 58.50%, respectively), while West Azerbaijan, Kurdistan, and Chahar Mahaal and Bakhtiari represented the lowest prevalence (39.53, 40.71, 40.73%, respectively) (Supplementary Table 2). Among males living in urban areas, Sistan and Baluchistan, Tehran, Alborz, and Golestan had a greater IPA prevalence. Females who lived in urban central, north-west, north-east, and south provinces had a higher prevalence of IPA. Furthermore, the IPA prevalence in rural areas for males was low in almost all provinces except for Alborz, which had a higher rate. Finally, Central and south-east provinces had a higher prevalence of IPA in rural areas for females (Figure 2).

### Physical activity domains in Iran

According to the physical activity domains, the age-standardized prevalence of no recreational activity was 79.40% (95% CI: 78.80–79.99%), no activity at work was 66.66% (95% CI: 65.99–67.32%), and no activity at transport was 49.40% (95% CI: 48.68–50.11%) in decreasing order for both-sexes combined. By sex, the largest domain in males and females was lacking recreational activities (70.78 and 86.35%, respectively). Moreover, the highest prevalence of physical inactivity attributable to work-, transport-, and recreational-domains was in the 75+ age group in both sexes combined. The lowest prevalence of physical inactivity was in 35–44 age group for work-domain and 18–24 age group for the transport- and recreational- domains (Table 3; Supplementary Figure 1).

There was a significant association between age, sex, education level, marital status, occupation, and wealth index with physical inactivity attributable to each domain (*p* < 0.001). The area of residence was only associated with the rate of work inactivity (*p* < 0.001), while it was not associated with transport (*p* = 0.095) and reactional (*p* = 0.804) inactivities. The prevalence of no physical activity at work was highest among people living in urban areas (72.32%), unemployed due to disability (85.26%), widow participants (81.15%), richest people (72.64%), and those who had 12 or more years of schooling (70.60%) (Supplementary Table 1). Alborz had the highest rate of physical inactivity attributable to work-, transport-, and recreational activities (84.13, 67.32, and 92.45%, respectively). In addition, South Khorasan showed the lowest rate of work inactivity (52.22%), and Kohgiluyeh and Boyer-Ahmad showed the lowest rate of transport and recreational inactivities (34.52 and 65.68%, respectively) (Supplementary Table 2).

### Physical activity intensity

The total METs attributable to vigorous-intensity physical activity was 3.88% (95% CI: 3.64–4.14%). The age-standardized percentage contribution of vigorous PA in total METs was higher in males than females (8.63 vs. 1.57%, respectively). Moreover, as was expected, the percentage of vigorous PA decreased with aging; the 18–24 years age group had the most considerable rate of vigorous physical activity in total METs, while 70–74 years old had the lowest rate (Table 3; Supplementary Figure 1).

The percentage contribution of vigorous PA in total MET was significantly associated with different demographic factors (*p* < 0.001) except for the wealth index (*p* = 0.38). Furthermore, participants who lived in rural areas (5.74%), had 12 or more years of schooling (5.02%), had freelance jobs or were self-employed (8.03%), had wealth index of class 3 (4.43%), and were single (8.43%) showed higher percentage contribution of vigorous PA in total MET (Table 1). Kohgiluyeh and Boyer-Ahmad represented the highest percentage contribution of vigorous PA in total MET (10.2%), while Yazd had the lowest percentage (1.39%) (Supplementary Table 2).

### Sedentary behaviors

The overall age-standardized prevalence of sedentary behaviors was 50.82% (95% CI: 50.11–51.53%) in both sexes in Iran. By sex, 49.42% of males and 51.95% of females had an age-standardized prevalence of sedentary lifestyle in daily life. Sedentary behavior was highest and lowest in 18–24 and 35–44 age groups, respectively (Table 2; Supplementary Figure 2).

The age-standardized prevalence of sedentary behaviors was associated with different demographic features (*p* < 0.001). The prevalence of sedentary behavior was highest among people living in urban areas (52.54%), unemployed individuals who do not seek work (67.01%), single participants (58.63%), richest people (56.60%), and those who had 12 or more years of schooling (55.57%) (Supplementary Table 1). Lorestan had the highest percentage of sedentary behaviors among both sexes (61.38%), while Kermanshah had the lowest percentage (31.28%) (Supplementary Table 2).

### Comorbidities

The prevalence of IPA was significantly higher in those with hypertension (55.17 vs. 49.36%; *p* < 0.001), diabetes mellitus (58.92 vs. 49.75%; *p* < 0.001), cardiovascular diseases (57.82 vs. 50.82%; *p* < 0.001), and dyslipidemia (52.26 vs. 48.15%; *p* = 0.001). Also, those with extreme obesity (i.e., BMI ≥ 40) had the highest prevalence of IPA (64.39%; 95% CI: 58.99–69.45%), while those with normal weight (i.e., 24.5 ≥ BMI > 18.5) had the lowest prevalence (49.12%; 95% CI: 47.93–50.32%) (Supplementary Table 1).

### Associates of insufficient physical activity in Iran

The crude and adjusted ORs of variables categorized by sex were summarized in Table 4. Among both sexes, living in urban area vs. rural area (adjusted OR: 1.44; 95% CI: 1.31–1.58), married and widow vs. single status (adjusted OR: 1.33; 95% CI: 1.16–1.53 and adjusted OR: 1.47; 95% CI: 1.21–1.80, respectively), unemployed due to disability and unemployed not seeking work vs. public sector employee (adjusted OR: 4.24; 95% CI: 1.57–11.44 and adjusted OR: 1.67; 95% CI: 1.01–2.75, respectively), and wealth index of class 3 vs. class 1 (adjusted OR: 1.15; 95% CI: 1.01–1.30) were significantly associated with higher rates of IPA. On the other hand, private Sector labor vs. public sector employee (adjusted OR: 0.54; 95% CI: 0.34–0.86), having basic health insurance vs. not having the insurance (adjusted 0.86; 95% CI: 0.75–0.99), and a BMI between 25 and 30 vs. BMI < 18.5 kg/m^2^ (adjusted OR: 0.79; 95% CI: 0.62–1) were significantly associated with lower rates of IPA. Considering the male gender, diabetes (adjusted OR: 1.68; 95% CI: 1.35–2.09), cardiovascular diseases (adjusted OR: 1.22; 95% CI: 1.03–1.43), and dyslipidemia (adjusted OR: 1.29; 95% CI: 1.11–1.51) significantly resulted in higher odds of IPA.

**Table 4:** Associates of insufficient physical activity in Iran.

**Variable**	**Category**	**Sex**	**OR crude**	**95% CI for crude OR**	**Adjusted OR^¥^**	**95% CI for adjusted OR**
Residential area (Reference: Rural area)	Urban	Female	**1.343**	(1.237, 1.457)	**1.44**	(1.311, 1.582)
		Male	**1.598**	(1.438, 1.775)	**1.74**	(1.549, 1.956)
		Total	**1.398**	(1.312, 1.489)	**1.44**	(1.311, 1.582)
Years of schooling (Reference: Zero year of schooling)	1–6	Female	**0.811**	(0.725, 0.907)	0.971	(0.854, 1.105)
		Male	**0.727**	(0.615, 0.859)	0.864	(0.718, 1.04)
		Total	**0.741**	(0.676, 0.812)	0.971	(0.854, 1.105)
	7–11	Female	**0.791**	(0.698, 0.895)	0.994	(0.851, 1.162)
		Male	**0.744**	(0.629, 0.88)	1.006	(0.823, 1.229)
		Total	**0.693**	(0.629, 0.764)	0.994	(0.851, 1.162)
	12 and over	Female	**0.72**	(0.649, 0.799)	0.922	(0.79, 1.078)
		Male	**0.668**	(0.574, 0.778)	0.845	(0.692, 1.032)
		Total	**0.63**	(0.58, 0.686)	0.922	(0.79, 1.078)
Marriage status (Reference: Single)	Married	Female	**1.277**	(1.145, 1.424)	**1.329**	(1.157, 1.528)
		Male	**1.439**	(1.282, 1.616)	1.18	(0.995, 1.399)
		Total	**1.411**	(1.305, 1.526)	**1.329**	(1.157, 1.528)
	Divorced/separated from partner	Female	1.037	(0.818, 1.314)	1.097	(0.849, 1.418)
		Male	1.38	(0.935, 2.037)	1.019	(0.676, 1.537)
		Total	**1.322**	(1.084, 1.612)	1.097	(0.849, 1.418)
	Widow	Female	**1.919**	(1.645, 2.24)	**1.474**	(1.21, 1.797)
		Male	**2.14**	(1.397, 3.276)	1.026	(0.625, 1.684)
		Total	**2.61**	(2.284, 2.981)	**1.474**	(1.21, 1.797)
Employment status (Reference: Public sector employee)	Retired	Female	1.147	(0.854, 1.541)	0.867	(0.633, 1.187)
		Male	0.991	(0.832, 1.179)	**0.788**	(0.644, 0.965)
		Total	0.952	(0.823, 1.101)	0.867	(0.633, 1.187)
	Unemployed due to disability	Female	**5.867**	(2.481, 13.874)	**4.245**	(1.575, 11.445)
		Male	**2.091**	(1.587, 2.757)	**1.71**	(1.25, 2.339)
		Total	**2.105**	(1.636, 2.71)	**4.245**	(1.575, 11.445)
	Unemployed seeker job	Female	1.174	(0.825, 1.67)	1.184	(0.807, 1.739)
		Male	0.813	(0.633, 1.045)	0.995	(0.757, 1.309)
		Total	0.898	(0.734, 1.1)	1.184	(0.807, 1.739)
	Unemployed not seeking work	Female	**1.601**	(1.009, 2.541)	**1.666**	(1.01, 2.748)
		Male	1.267	(0.9, 1.783)	1.219	(0.844, 1.762)
		Total	**1.368**	(1.041, 1.798)	**1.666**	(1.01, 2.748)
	Public sector labor	Female	0.611	(0.301, 1.241)	0.564	(0.273, 1.166)
		Male	**0.622**	(0.418, 0.926)	**0.564**	(0.372, 0.855)
		Total	**0.591**	(0.419, 0.834)	0.564	(0.273, 1.166)
	Private sector employee	Female	0.862	(0.641, 1.16)	0.853	(0.627, 1.161)
		Male	0.85	(0.65, 1.113)	0.85	(0.646, 1.118)
		Total	0.886	(0.728, 1.079)	0.853	(0.627, 1.161)
	Private sector labor	Female	**0.576**	(0.371, 0.894)	**0.542**	(0.341, 0.86)
		Male	**0.656**	(0.522, 0.826)	**0.674**	(0.528, 0.861)
		Total	**0.604**	(0.495, 0.738)	**0.542**	(0.341, 0.86)
	Freelance job or self-employed	Female	0.923	(0.711, 1.198)	0.823	(0.624, 1.086)
		Male	**0.796**	(0.679, 0.932)	**0.814**	(0.686, 0.966)
		Total	**0.751**	(0.659, 0.855)	0.823	(0.624, 1.086)
	Unpaid work	Female	**1.292**	(1.065, 1.567)	1.066	(0.863, 1.317)
		Male	**0.732**	(0.583, 0.92)	1.081	(0.823, 1.418)
		Total	**1.482**	(1.313, 1.671)	1.066	(0.863, 1.317)
Basic health insurance (Reference: No basic health insurance)	Yes	Female	**0.87**	(0.764, 0.99)	**0.86**	(0.748, 0.989)
		Male	**0.843**	(0.737, 0.964)	**0.798**	(0.689, 0.925)
		Total	**0.904**	(0.825, 0.992)	**0.86**	(0.748, 0.989)
Complementary insurance (Reference: No complementary insurance)	Yes	Female	**1.137**	(1.048, 1.233)	1.05	(0.955, 1.153)
		Male	1.074	(0.976, 1.181)	0.929	(0.824, 1.048)
		Total	**1.1**	(1.035, 1.169)	1.05	(0.955, 1.153)
Wealth index [Reference: Level 1 (poorest)]	Level 2	Female	1.001	(0.892, 1.124)	1.001	(0.887, 1.129)
		Male	1.025	(0.887, 1.186)	1.009	(0.866, 1.176)
		Total	1.005	(0.919, 1.1)	1.001	(0.887, 1.129)
	Level 3	Female	1.088	(0.965, 1.227)	**1.146**	(1.011, 1.299)
		Male	0.977	(0.846, 1.128)	1.034	(0.888, 1.204)
		Total	1	(0.913, 1.095)	**1.146**	(1.011, 1.299)
	Level 4	Female	1.03	(0.914, 1.16)	1.067	(0.938, 1.213)
		Male	0.915	(0.793, 1.055)	0.917	(0.784, 1.074)
		Total	0.939	(0.859, 1.028)	1.067	(0.938, 1.213)
	Level 5 (richest)	Female	0.89	(0.791, 1.001)	0.942	(0.823, 1.079)
		Male	0.978	(0.85, 1.124)	0.949	(0.806, 1.116)
		Total	**0.886**	(0.811, 0.968)	0.942	(0.823, 1.079)
Hypertension (Reference: Blood pressure below 140/90)	Blood pressure above 140/90	Female	**1.289**	(1.193, 1.393)	1.098	(0.996, 1.211)
		Male	**1.226**	(1.117, 1.345)	1.03	(0.927, 1.144)
		Total	**1.263**	(1.191, 1.339)	1.098	(0.996, .211)
Diabetes (Reference: No)	Yes	Female	**1.266**	(1.083, 1.48)	1.168	(0.978, 1.394)
		Male	**1.761**	(1.433, 2.164)	**1.678**	(1.346, 2.092)
		Total	**1.449**	(1.279, 1.641)	1.168	(0.978, 1.394)
Cardiovascular diseases (Reference: No)	Yes	Female	**1.478**	(1.253, 1.743)	1.122	(0.939, 1.341)
		Male	**1.448**	(1.245, 1.683)	**1.216**	(1.031, 1.434)
		Total	**1.326**	(1.189, 1.48)	1.122	(0.939, 1.341)
Body mass index	18.5–24.9	Female	0.931	(0.754, 1.149)	0.848	(0.672, 1.07)
		Male	1.209	(0.951, 1.537)	1.19	(0.918, 1.543)
		Total	0.999	(0.856, 1.165)	0.848	(0.672, 1.07)
	25–29.9	Female	0.891	(0.723, 1.098)	**0.788**	(0.623, 0.997)
		Male	**1.299**	(1.023, 1.65)	1.217	(0.936, 1.582)
		Total	1.038	(0.891, 1.21)	**0.788**	(0.623, 0.997)
	30–34.9	Female	0.907	(0.732, 1.124)	0.784	(0.615, 1)
		Male	**1.58**	(1.223, 2.04)	**1.483**	(1.122, 1.959)
		Total	**1.198**	(1.021, 1.406)	0.784	(0.615, 1)
	35–39.9	Female	1.247	(0.977, 1.593)	1.063	(0.809, 1.397)
		Male	**1.685**	(1.201, 2.363)	**1.558**	(1.088, 2.231)
		Total	**1.614**	(1.333, 1.953)	1.063	(0.809, 1.397)
	40+	Female	**1.396**	(1.014, 1.923)	1.28	(0.902, 1.815)
		Male	1.753	(0.945, 3.252)	1.771	(0.932, .366)
		Total	**1.87**	(1.426, 2.453)	1.28	(0.902, 1.815)
Dyslipidemia (Reference: no)	Yes	Female	1.008	(0.887, 1.146)	0.996	(0.87, 1.14)
		Male	**1.259**	(1.084, 1.462)	**1.292**	(1.108, 1.507)
		Total	**1.179**	(1.071, 1.297)	0.996	(0.87, 1.14)

OR, odds ratio; CI, confidence interval. Bold values represent the statistical significance.

¥ The model was adjusted for the effects of socioeconomic factors, including residential areas, years of schooling, wealth index, age, marital status, and occupation.

## Discussion

In this descriptive study using data from a national survey, we found that more than half of the Iranian population had IPA and its prevalence is higher among females. Moreover, the IPA was primarily due to insufficient recreational activities. Overall, living in the urban area compared to rural area, married vs. single status, and wealth index of class 3 vs. class 1 were substantially associated with higher rates of IPA.

The results of STEPS 2016 study revealed that the mean prevalence of IPA among both sexes in Iran was 54.7% (95%CI: 54.0–55.3%), while our study showed a prevalence of 51.30% (95% CI: 50.62–51.98%), which is a reduction (9). On the other hand, the prevalence of sedentary behaviors was lower in 2016 in comparison with 2021 (33.60 vs. 49.97%) (9). Given the outbreak of the COVID-19 pandemic and as a consequence of lockdown policies aimed at controlling the spread of the disease, the level of IPA was dramatically increased and the world faces a novel problem in exacerbating the pandemic of inactivity. A study that included step counts as an indicator of physical activity showed that the step counts significantly decreased after the announcement of COVID-19 as a global pandemic (26). Thus, the elevation in the level of IPA prevalence in Iran from 2016 to 2021 could be a result of the impact of COVID-19 on people's lifestyles. In the present study, a high proportion of participants had unpaid work, which can be a result of COVID-19 pandemic lockdown and quarantine. Moreover, IPA had a higher prevalence among females, those aged above 75 years, and individuals with obesity. Therefore, the findings should be considered by policymakers for potential future pandemics that women, elderly, unemployed population, and those with underlying diseases, in particular obesity should have a priority for plans to encourage for more physical activity.

Findings of a population-based survey on 1.9 million participants across 168 countries showed that the global age-standardized prevalence of IPA was 27.5% [95% uncertainty interval (UI): 25.0–32.2%] in 2016 (27), which was lower than its age-standardized prevalence for Iran in 2021 (50.35%). In addition, over 2001–2016, the global age-standardized prevalence of IPA decreased; however, it was not significant (27). The prevalence of IPA based on data from STEPS survey in neighboring Arab countries showed that the prevalence ranged from 5.2% in Jordan to 67.6% in Saudi Arabia, but most countries like Iraq (47.0%), Libya (43.9%), Palestine (46.5%), and Qatar (45.9%) had the prevalence close to Iran (28). The article by Dumith et al. (29) published in 2011, showed a positive association between the human development index and prevalence of IPA (ρ = 0.27). However, we found a significant lower prevalence of IPA in the wealthiest quintile (*p* = 0.015). The difference might be as a result of the year of conduction of the studies. At subnational level, Yazd, Alborz, and Sistan and Baluchistan provinces had a higher prevalence of IPA in 2021. However, Bushehr, Yazd, and Ardebil were the top-ranked provinces with the highest IPA prevalence in 2016 (9).

There was a significant sex disparity in physical activity in which women had a higher rate of IPA. In accordance with our results, STEPS 2016 also showed a higher prevalence of IPA among females than males (61.9 vs. 45.3%) (9). In this regard, a similar study using the WHO STEPS database revealed a higher prevalence of IPA in women than men in Oman in 2017 (50.5 vs. 30.1%) (30). Moreover, the global age-standardized prevalence of IPA was 31.7% in females and 23.4% in males in 2016 (27). Meanwhile, in line with our findings, with a rate of 68.1% in 2021, the elderly aged above 75 years old had the highest prevalence of IPA in 2016 (73.8%) (9). Previous studies also revealed a higher prevalence of IPA among older adults (31–33).

In 2016, the lowest domain of physical activities was recreation (12.8%), and the highest was work (53.7%) (9). Similarly, the lack of recreational activities was the biggest contributor of IPA in 2021.

Given the fact that the prevalence of IPA and no recreational activities is high in Iran, health policymakers should focus on the reduction of obstacles and facilitate access to sports equipment. In this regard, there are different barriers for physical activity among the elderly in Iran and the world, which are categorized into three major categories, including interpersonal (e.g., having no companion, having no professional guidance, family responsibilities, and social pressures), intrapersonal (e.g., physical problems, time restrictions, lack of interest, laziness, financial cost, security concerns, and fear of falling), and environmental issues (e.g., traffic, weather, and physical barriers to walking) (34).

Our study showed that urbanization had a strong association with IPA (adjusted OR: 1.44; 95% CI: 1.31–1.58), which was in accordance with the STEPS 2016 study (adjusted OR: 1.69; 95% CI: 1.52–1.88) (9). A slight difference in the odds of IPA in urban areas in 2021 compared with 2016 could be a result of using different covariates for adjustment in the logistic regression analysis. In 2016, the analysis included not only sociodemographic and lifestyle-related variables but also metabolic risk factors in the analysis (9). Furthermore, a study on older adults in the Northwest of Iran suggested comorbidities as a significant predictor of physical activity (β = −22.15, *p* = 0.001) (35). In this regard, we found that participants with diabetes, hypertension, cardiovascular diseases, and dyslipidemia had 9.17%, 5.81, 7.00, and 4.11% higher rates of IPA, respectively.

According to the SDG 3.4 target, a reduction by one-third in premature mortality from NCDs through prevention and treatment and promoting mental health and wellbeing is expected by 2030 (6). Up to this year, a 15% relative reduction in the global prevalence of physical inactivity in adults and in adolescents was set as a global action plan (7). In this case, WHO provided policymakers with a list of overarching/enabling policy actions, the most cost-effective interventions, and other recommended interventions to address NCDs. Among these interventions, the most cost-effective and feasible implementation options were considered as “best buys.” Considering physical inactivity as a key modifiable risk factor for NCDs, the “best buys” were to implement community-wide public education and awareness campaigns for physical activity, which includes a mass media campaign combined with other community-based education, motivational and environmental programs aimed at supporting behavioral change of physical activity levels (36). A wide range of actions is required to facilitate the realization of public health goals in terms of reducing physical inactivity in Iran. Inter-organizational collaboration is required in the first step. Moreover, vulnerable groups like women and the elderly population should be given greater priority. It is necessary to establish safe and culturally acceptable opportunities for women to have physical activity. Also, financial resources should be allocated to increase physical activity (37, 38).

Our study is a large-scale observational study at the national and sub-national levels and provides the most up-to-date data on the patterns of physical activity in Iran during the COVID-19 pandemic. Nevertheless, we acknowledge that it has several limitations. Firstly, we only included adults above 18 years old, so the data on the prevalence of physical activity among children and adolescents has not been assessed in the present study. Secondly, since we have used self-reported data for most demographic factors, socioeconomic features, and physical activity, they are susceptible to recall bias or over-or under-estimation. Thirdly, the validity and reliability of GPAQ have not been evaluated in rural areas, so it might lead to not valid data. Fourthly, eliminating the samples without anthropometry and laboratory information could bias the results, which should be considered in the interpretation of the results. Finally, the study's cross-sectional nature is unable to represent a causal relationship.

## Conclusion

Our study showed that the prevalence of IPA is high in Iran, especially among females and the elderly population. Increased physical activity in urban areas, among females, and provinces with lower socioeconomic status should be implemented to achieve the global and national goal of reduction in IPA based on the action plans for controlling IPA. However, those 75 years old and above female would have difficulty in physical activities and it should be considered in planning for their physical activity patterns. Further regular large-scale cohort studies are required to evaluate the causal relationship between different underlying conditions and the rate of IPA and to establish practical methods for assigning sufficient physical activities for individuals based on demographical and clinical characteristics. Moreover, preparing the results of physical activity patterns before and after the COVID-19 pandemic can be considered in the next studies. Also, this study only provides the associated factors with IPA. So, further regular large-scale cohort studies are required to evaluate the causal relationship between different underlying conditions and the rate of IPA and to establish practical methods for assigning sufficient physical activities for individuals based on demographic and clinical characteristics.

## Data availability statement

The raw data supporting the conclusions of this article will be made available by the authors, without undue reservation.

## Ethics statement

The studies involving human participants were reviewed and approved by Ethical Committee of the National Institute of Health Research (NIHR), Tehran University of Medical Sciences, Tehran, Iran (ID: IR.TUMS.NIHR.REC.1398.006). The patients/participants provided their written informed consent to participate in this study.

## Author contributions

General design of the paper: FF, NR, and SD. Designing of methods: FF, NR, NA, AG, MNa, MY, and EG. Data analysis: FF, NA, AG, MNa, MY, and EG. Writing the primary draft: SN, M-MR, MNo, and AG. Manuscript revision: FF, NR, NF, SN, M-MR, MNo, AG, MD, and MA-K. Administrative process: EA and YF. All authors have read and approved the final manuscript.

## Supplementary material

Supplementary Figure 1 Three pie charts which represent the % prevalence of each physical activity domain for females; males; and both sexes among Iranian population in 2021.

Supplementary Figure 2 Prevalence of sedentary lifestyle among Iranian population in 2021, by sex and age.

Supplementary Table 1 Prevalence of physical activity domains, sedentary behaviors, and insufficient physical activity among women and men of various underlying conditions. MET, Metabolic equivalent of task; CI, confidence interval.

Supplementary Table 2 Prevalence of physical activity domains, sedentary behaviors, and insufficient physical activity among women and men, by province. MET, Metabolic equivalent of task; CI, confidence interval.

Supplementary Presentation 1 The map of Iran provinces.
