# Supplementary figures and images for: Physical activity pattern in Iran: Findings from STEPS 2021
Source: Front Public Health. 2023 Jan 4;10:1036219. doi: 10.3389/fpubh.2022.1036219 (PMC9846211; doi:10.3389/fpubh.2022.1036219)

**Female**

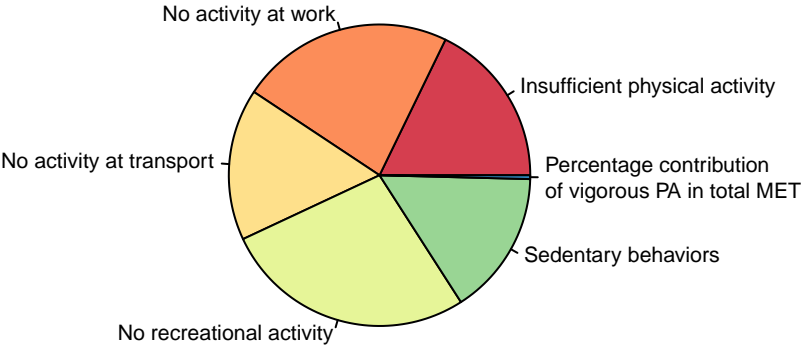

**Male**

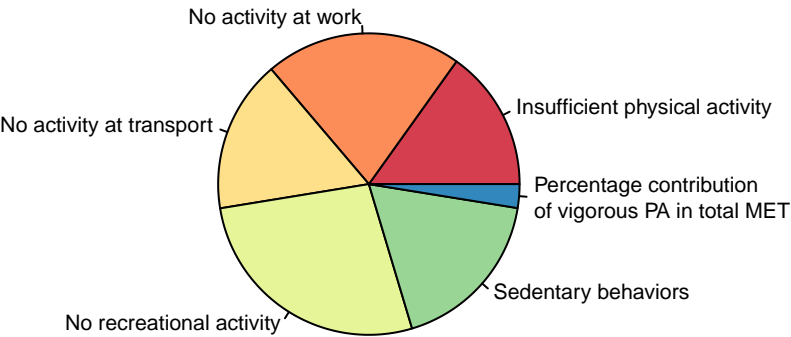

**Total**

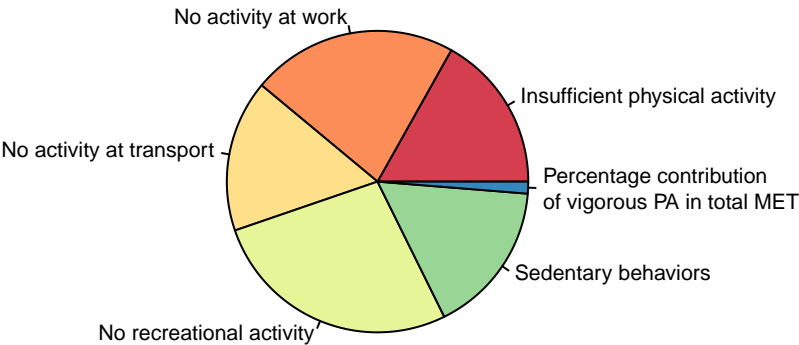

Supplement: Supplementary Figure 1 — Three pie charts which represent the % prevalence of each physical activity domain for females; males; and both sexes among Iranian population in 2021. [file Image_1.pdf]

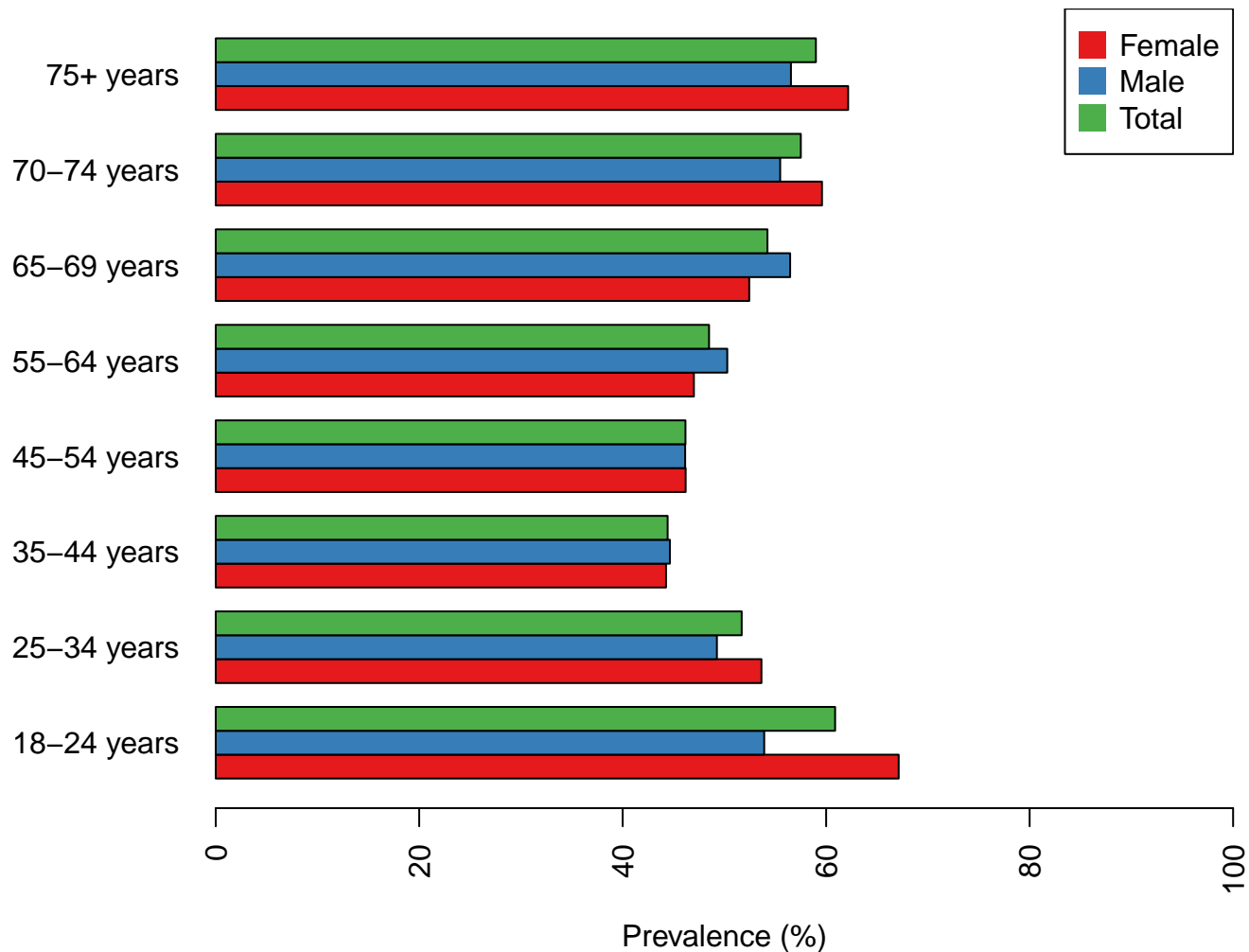

Supplement: Supplementary Figure 2 — Prevalence of sedentary lifestyle among Iranian population in 2021, by sex and age. [file Image_2.pdf]
