# Supplementary material for: Physical activity pattern in Iran: Findings from STEPS 2021
Source: Front Public Health. 2023 Jan 4;10:1036219. doi: 10.3389/fpubh.2022.1036219 (PMC9846211; doi:10.3389/fpubh.2022.1036219)
Supplement: Supplementary Table 1 — Prevalence of physical activity domains, sedentary behaviors, and insufficient physical activity among women and men of various underlying conditions. MET, Metabolic equivalent of task; CI, confidence interval. [file Table_1.DOCX]

**Table S1.** Prevalence of physical activity domains, sedentary behaviors, and insufficient physical activity among women and men of various underlying conditions.

| **Variable** | **Category** | | **Insufficient physical activity** | | **No activity at work** | | **No activity at transport** | | **No recreational activity** | | **Sedentary behaviors** | | **Contribution of vigorous physical activity in total MET** | |
| --- | --- | --- | --- | --- | --- | --- | --- | --- | --- | --- | --- | --- | --- | --- |
|  |  |  | **N (%)** | **95% CI** | **N (%)** | **95% CI** | **N (%)** | **95% CI** | **N (%)** | **95% CI** | **N (%)** | **95% CI** | **N (%)** | **95% CI** |
| Hypertension | Yes | Female | 3065 (61.93%) | (60.43 , 63.42)% | 3840 (75.42%) | (74.1 , 76.7)% | 2904 (57.48%) | (55.97 , 58.99)% | 4760 (92.79%) | (91.95 , 93.55)% | 2600 (51.57%) | (50.04 , 53.1)% | 31 (0.6%) | (0.41 , 0.89)% |
|  |  | Male | 1527 (45.27%) | (43.41 , 47.15)% | 2475 (64.5%) | (62.81 , 66.15)% | 1723 (43.81%) | (42.07 , 45.56)% | 3151 (81.05%) | (79.63 , 82.4)% | 2081 (53.21%) | (51.45 , 54.97)% | 176 (4.55%) | (3.87 , 5.35)% |
|  |  | Total | 4592 (55.17%) | (53.99 , 56.35)% | 6315 (70.71%) | (69.66 , 71.73)% | 4627 (51.58%) | (50.43 , 52.74)% | 7911 (87.73%) | (86.95 , 88.47)% | 4681 (52.28%) | (51.12 , 53.43)% | 207 (2.31%) | (1.98 , 2.68)% |
|  | p-value | | <0.001 | | <0.001 | | <0.001 | | <0.001 | | 0.168 | | <0.001 | |
|  | No | Female | 5442 (55.8%) | (54.71 , 56.89)% | 7485 (73.96%) | (73 , 74.89)% | 5118 (50.69%) | (49.61 , 51.78)% | 8768 (85.89%) | (85.11 , 86.64)% | 5072 (49.95%) | (48.87 , 51.03)% | 163 (1.71%) | (1.43 , 2.04)% |
|  |  | Male | 2728 (40.29%) | (39.02 , 41.58)% | 4628 (55.67%) | (54.5 , 56.83)% | 3875 (46.07%) | (44.89 , 47.25)% | 6075 (72.34%) | (71.28 , 73.38)% | 4087 (47.76%) | (46.58 , 48.94)% | 740 (8.19%) | (7.57 , 8.85)% |
|  |  | Total | 8170 (49.36%) | (48.52 , 50.2)% | 12113 (65.66%) | (64.9 , 66.4)% | 8993 (48.6%) | (47.8 , 49.39)% | 14843 (79.74%) | (79.09 , 80.37)% | 9159 (48.96%) | (48.16 , 49.75)% | 903 (4.65%) | (4.33 , 5)% |
|  | p-value | | <0.001 | | <0.001 | | <0.001 | | <0.001 | | 0.007 | | <0.001 | |
|  | p-value (between groups and both sexes) | | <0.001 | | <0.001 | | <0.001 | | <0.001 | | <0.001 | | <0.001 | |
| Diabetes mellitus | No | Female | 4660 (56.71%) | (55.21 , 58.2)% | 6192 (72.49%) | (71.18 , 73.77)% | 4481 (53.09%) | (51.6 , 54.57)% | 7819 (88.34%) | (87.33 , 89.29)% | 4150 (47.84%) | (46.34 , 49.33)% | 116 (1.39%) | (1.04 , 1.86)% |
|  |  | Male | 2129 (39.79%) | (37.95 , 41.66)% | 3547 (55.02%) | (53.35 , 56.67)% | 2989 (45.75%) | (44.08 , 47.44)% | 5144 (75.12%) | (73.59 , 76.59)% | 3327 (48.78%) | (47.1 , 50.47)% | 525 (7.3%) | (6.51 , 8.18)% |
|  |  | Total | 6789 (49.75%) | (48.56 , 50.94)% | 9739 (64.67%) | (63.62 , 65.7)% | 7470 (49.81%) | (48.69 , 50.92)% | 12963 (82.42%) | (81.53 , 83.29)% | 7477 (48.26%) | (47.14 , 49.38)% | 641 (4.04%) | (3.62 , 4.5)% |
|  | p-value | | <0.001 | | <0.001 | | <0.001 | | <0.001 | | 0.411 | | <0.001 | |
|  | Yes | Female | 869 (62.38%) | (58.95 , 65.7)% | 1105 (77.36%) | (74.48 , 80)% | 806 (54.77%) | (51.29 , 58.2)% | 1358 (93.73%) | (91.86 , 95.19)% | 766 (51.92%) | (48.44 , 55.38)% | 9 (0.93%) | (0.41 , 2.13)% |
|  |  | Male | 459 (53.78%) | (49.03 , 58.46)% | 666 (69.88%) | (65.73 , 73.73)% | 441 (41.7%) | (37.44 , 46.1)% | 850 (84.92%) | (81.37 , 87.89)% | 544 (55.87%) | (51.34 , 60.29)% | 26 (2.65%) | (1.56 , 4.46)% |
|  |  | Total | 1328 (58.92%) | (56.12 , 61.67)% | 1771 (74.2%) | (71.8 , 76.46)% | 1247 (49.25%) | (46.52 , 51.99)% | 2208 (90.01%) | (88.19 , 91.58)% | 1310 (53.58%) | (50.81 , 56.33)% | 35 (1.66%) | (1.06 , 2.58)% |
|  | p-value | | 0.004 | | 0.002 | | <0.001 | | <0.001 | | 0.174 | | 0.029 | |
|  | p-value (between groups and both sexes) | | <0.001 | | <0.001 | | 0.717 | | <0.001 | | <0.001 | | <0.001 | |
| Cardiovascular diseases | No | Female | 7986 (57.38%) | (56.47 , 58.28)% | 10711 (74.35%) | (73.56 , 75.13)% | 7510 (52.34%) | (51.44 , 53.25)% | 12759 (87.83%) | (87.22 , 88.42)% | 7217 (50.07%) | (49.17 , 50.98)% | 188 (1.34%) | (1.15 , 1.57)% |
|  |  | Male | 3819 (41.11%) | (40.01 , 42.21)% | 6407 (56.97%) | (55.97 , 57.98)% | 5156 (45.42%) | (44.4 , 46.43)% | 8381 (73.87%) | (72.96 , 74.75)% | 5599 (48.55%) | (47.53 , 49.57)% | 901 (7.53%) | (7.02 , 8.09)% |
|  |  | Total | 11805 (50.82%) | (50.12 , 51.53)% | 17118 (66.72%) | (66.09 , 67.35)% | 12666 (49.3%) | (48.63 , 49.98)% | 21140 (81.7%) | (81.17 , 82.22)% | 12816 (49.4%) | (48.73 , 50.08)% | 1089 (4.06%) | (3.8 , 4.33)% |
|  | p-value | | <0.001 | | <0.001 | | <0.001 | | <0.001 | | 0.029 | | <0.001 | |
|  | Yes | Female | 536 (66.55%) | (62.87 , 70.03)% | 633 (76.21%) | (72.89 , 79.23)% | 524 (63.21%) | (59.5 , 66.77)% | 792 (94.57%) | (92.57 , 96.05)% | 468 (57.07%) | (53.31 , 60.76)% | 6 (0.83%) | (0.35 , 1.95)% |
|  |  | Male | 452 (50.26%) | (46.67 , 53.85)% | 724 (74.45%) | (71.4 , 77.28)% | 462 (44.96%) | (41.56 , 48.41)% | 873 (87.54%) | (85.09 , 89.64)% | 587 (58.76%) | (55.32 , 62.12)% | 20 (1.76%) | (1.07 , 2.88)% |
|  |  | Total | 988 (57.82%) | (55.22 , 60.38)% | 1357 (75.24%) | (73.03 , 77.33)% | 986 (53.18%) | (50.63 , 55.72)% | 1665 (90.71%) | (89.13 , 92.08)% | 1055 (58%) | (55.47 , 60.49)% | 26 (1.34%) | (0.87 , 2.06)% |
|  | p-value | | <0.001 | | 0.429 | | <0.001 | | <0.001 | | 0.513 | | 0.13 | |
|  | p-value (between groups and both sexes) | | <0.001 | | <0.001 | | 0.004 | | <0.001 | | <0.001 | | <0.001 | |
| BMI | <18.5 | Female | 290 (59.3%) | (54.43 , 63.99)% | 371 (75.87%) | (71.66 , 79.64)% | 268 (54.3%) | (49.43 , 59.1)% | 427 (85.57%) | (81.74 , 88.71)% | 282 (56.99%) | (52.21 , 61.64)% | 8 (2.1%) | (1.04 , 4.22)% |
|  |  | Male | 145 (35.67%) | (30.55 , 41.13)% | 250 (52.76%) | (47.67 , 57.79)% | 208 (42.9%) | (37.99 , 47.95)% | 355 (74.31%) | (69.53 , 78.58)% | 243 (49.65%) | (44.61 , 54.69)% | 28 (6.06%) | (4.02 , 9.02)% |
|  |  | Total | 435 (49.16%) | (45.52 , 52.81)% | 621 (64.92%) | (61.56 , 68.13)% | 476 (48.87%) | (45.38 , 52.38)% | 782 (80.24%) | (77.24 , 82.93)% | 525 (53.52%) | (50.06 , 56.95)% | 36 (3.98%) | (2.79 , 5.64)% |
|  | p-value | | <0.001 | | <0.001 | | 0.001 | | <0.001 | | 0.04 | | 0.007 | |
|  | 18.5-24.9 | Female | 2425 (57.56%) | (55.91 , 59.19)% | 3268 (74.52%) | (73.08 , 75.92)% | 2252 (51.39%) | (49.74 , 53.03)% | 3867 (86.57%) | (85.38 , 87.68)% | 2307 (52.11%) | (50.47 , 53.74)% | 64 (1.68%) | (1.25 , 2.25)% |
|  |  | Male | 1563 (40.09%) | (38.42 , 41.8)% | 2668 (55.03%) | (53.5 , 56.56)% | 2143 (43.24%) | (41.71 , 44.78)% | 3569 (72.84%) | (71.45 , 74.19)% | 2389 (48.04%) | (46.49 , 49.59)% | 441 (8.33%) | (7.52 , 9.21)% |
|  |  | Total | 3988 (49.12%) | (47.93 , 50.32)% | 5936 (64.26%) | (63.19 , 65.31)% | 4395 (47.09%) | (45.97 , 48.22)% | 7436 (79.34%) | (78.41 , 80.24)% | 4696 (49.97%) | (48.84 , 51.09)% | 505 (5.18%) | (4.7 , 5.71)% |
|  | p-value | | <0.001 | | <0.001 | | <0.001 | | <0.001 | | <0.001 | | <0.001 | |
|  | 25-29.9 | Female | 3038 (56.49%) | (55.01 , 57.95)% | 4162 (74.66%) | (73.38 , 75.89)% | 2842 (51.04%) | (49.58 , 52.5)% | 4954 (88.19%) | (87.21 , 89.1)% | 2713 (48.78%) | (47.32 , 50.24)% | 73 (1.29%) | (1 , 1.66)% |
|  |  | Male | 1682 (41.83%) | (40.17 , 43.51)% | 2852 (60.04%) | (58.5 , 61.55)% | 2176 (45.68%) | (44.12 , 47.25)% | 3621 (75.46%) | (74.09 , 76.78)% | 2375 (48.43%) | (46.86 , 50)% | 338 (6.8%) | (6.06 , 7.63)% |
|  |  | Total | 4720 (50.1%) | (48.98 , 51.21)% | 7014 (67.86%) | (66.87 , 68.84)% | 5018 (48.55%) | (47.48 , 49.62)% | 8575 (82.27%) | (81.44 , 83.07)% | 5088 (48.61%) | (47.55 , 49.68)% | 411 (3.85%) | (3.46 , 4.28)% |
|  | p-value | | <0.001 | | <0.001 | | <0.001 | | <0.001 | | 0.749 | | <0.001 | |
|  | 30-34.9 | Female | 1820 (56.93%) | (55.01 , 58.83)% | 2400 (72.85%) | (71.13 , 74.5)% | 1752 (53.82%) | (51.92 , 55.71)% | 2941 (89.46%) | (88.21 , 90.58)% | 1566 (48%) | (46.1 , 49.9)% | 31 (0.97%) | (0.66 , 1.43)% |
|  |  | Male | 678 (46.65%) | (43.85 , 49.47)% | 1069 (63.27%) | (60.69 , 65.77)% | 841 (49.33%) | (46.69 , 51.98)% | 1340 (78.8%) | (76.55 , 80.89)% | 919 (54.13%) | (51.48 , 56.75)% | 90 (5.04%) | (4.01 , 6.32)% |
|  |  | Total | 2498 (53.66%) | (52.07 , 55.24)% | 3469 (69.57%) | (68.14 , 70.97)% | 2593 (52.29%) | (50.74 , 53.82)% | 4281 (85.81%) | (84.69 , 86.86)% | 2485 (50.09%) | (48.55 , 51.63)% | 121 (2.36%) | (1.94 , 2.88)% |
|  | p-value | | <0.001 | | <0.001 | | 0.007 | | <0.001 | | <0.001 | | <0.001 | |
|  | 35-39.9 | Female | 684 (64.5%) | (61.21 , 67.67)% | 838 (77.15%) | (74.25 , 79.82)% | 651 (60.08%) | (56.73 , 63.33)% | 979 (89.35%) | (87.06 , 91.28)% | 581 (53.27%) | (49.91 , 56.59)% | 13 (1.19%) | (0.66 , 2.16)% |
|  |  | Male | 152 (48.39%) | (42.21 , 54.61)% | 217 (65.19%) | (59.35 , 70.6)% | 186 (52.61%) | (46.62 , 58.53)% | 281 (82.97%) | (77.94 , 87.04)% | 195 (59.14%) | (53.18 , 64.85)% | 14 (4.43%) | (2.51 , 7.72)% |
|  |  | Total | 836 (60.94%) | (58.01 , 63.79)% | 1055 (74.34%) | (71.75 , 76.77)% | 837 (58.38%) | (55.45 , 61.24)% | 1260 (87.89%) | (85.82 , 89.69)% | 776 (54.59%) | (51.66 , 57.49)% | 27 (1.94%) | (1.28 , 2.92)% |
|  | p-value | | <0.001 | | <0.001 | | 0.033 | | 0.006 | | 0.097 | | 0.001 | |
|  | 40+ | Female | 225 (67.04%) | (61.31 , 72.31)% | 264 (74.38%) | (68.95 , 79.14)% | 237 (68.77%) | (63.08 , 73.95)% | 331 (94.78%) | (91.58 , 96.81)% | 207 (61.18%) | (55.38 , 66.68)% | 4 (1.03%) | (0.32 , 3.25)% |
|  |  | Male | 29 (50.73%) | (35.78 , 65.54)% | 43 (67.58%) | (53.8 , 78.86)% | 40 (58.04%) | (43.9 , 70.98)% | 51 (75.36%) | (61.55 , 85.39)% | 40 (48.26%) | (34.78 , 62.01)% | 5 (6.42%) | (2.22 , 17.16)% |
|  |  | Total | 254 (64.39%) | (58.99 , 69.45)% | 307 (72.63%) | (67.67 , 77.09)% | 277 (66.67%) | (61.46 , 71.5)% | 382 (90.99%) | (87.5 , 93.58)% | 247 (59.64%) | (54.31 , 64.76)% | 9 (1.94%) | (0.92 , 4.05)% |
|  | p-value | | 0.019 | | 0.105 | | 0.073 | | <0.001 | | 0.204 | | 0.006 | |
|  | p-value (between groups and both sexes) | | <0.001 | | <0.001 | | <0.001 | | <0.001 | | <0.001 | | <0.001 | |
| Dyslipidemia | No | Female | 1347 (57.39%) | (54.68 , 60.06)% | 1798 (73.94%) | (71.58 , 76.16)% | 1278 (50.19%) | (47.5 , 52.88)% | 2264 (88.7%) | (86.85 , 90.33)% | 1203 (47.81%) | (45.13 , 50.51)% | 36 (1.82%) | (0.96 , 3.43)% |
|  |  | Male | 800 (38.13%) | (35.32 , 41.01)% | 1312 (52.39%) | (49.84 , 54.93)% | 1090 (41.82%) | (39.3 , 44.4)% | 2071 (75.93%) | (73.51 , 78.18)% | 1246 (46.55%) | (43.97 , 49.15)% | 205 (7.5%) | (6.24 , 9)% |
|  |  | Total | 2147 (48.15%) | (46.16 , 50.15)% | 3110 (62.72%) | (60.98 , 64.44)% | 2368 (45.84%) | (43.99 , 47.69)% | 4335 (82.05%) | (80.5 , 83.5)% | 2449 (47.16%) | (45.3 , 49.02)% | 241 (4.78%) | (3.95 , 5.78)% |
|  | p-value | | <0.001 | | <0.001 | | <0.001 | | <0.001 | | 0.508 | | <0.001 | |
|  | Yes | Female | 4177 (57.59%) | (56 , 59.17)% | 5496 (72.93%) | (71.54 , 74.28)% | 4005 (54.4%) | (52.83 , 55.97)% | 6907 (89.26%) | (88.2 , 90.23)% | 3710 (48.59%) | (46.99 , 50.19)% | 89 (1.16%) | (0.9 , 1.5)% |
|  |  | Male | 1789 (43.68%) | (41.51 , 45.88)% | 2899 (59.47%) | (57.53 , 61.38)% | 2337 (46.91%) | (44.93 , 48.9)% | 3917 (76.73%) | (74.98 , 78.39)% | 2622 (51.38%) | (49.39 , 53.36)% | 347 (6.26%) | (5.43 , 7.21)% |
|  |  | Total | 5966 (52.26%) | (50.95 , 53.57)% | 8395 (67.37%) | (66.22 , 68.5)% | 6342 (51.31%) | (50.06 , 52.55)% | 10824 (84.08%) | (83.12 , 84.99)% | 6332 (49.74%) | (48.49 , 50.99)% | 436 (3.27%) | (2.88 , 3.7)% |
|  | p-value | | <0.001 | | <0.001 | | <0.001 | | <0.001 | | 0.032 | | <0.001 | |
|  | p-value (between groups and both sexes) | | 0.001 | | <0.001 | | <0.001 | | 0.022 | | 0.024 | | 0.001 | |

Abbreviations: MET: Metabolic equivalent of task; CI: confidence interval.
