# Supplementary material for: Physical activity pattern in Iran: Findings from STEPS 2021
Source: Front Public Health. 2023 Jan 4;10:1036219. doi: 10.3389/fpubh.2022.1036219 (PMC9846211; doi:10.3389/fpubh.2022.1036219)
Supplement: Supplementary Table 2 — Prevalence of physical activity domains, sedentary behaviors, and insufficient physical activity among women and men, by province. MET, Metabolic equivalent of task; CI, confidence interval. [file Table_2.DOCX]

**Table S2.** Prevalence of physical activity domains, sedentary behaviors, and insufficient physical activity among women and men, by province.

| **Provinces** | **Category** | **Insufficient physical activity** | | **No activity at work** | | **No activity at transport** | | **No recreational activity** | | **Sedentary behaviors** | | **Contribution of vigorous physical activity in total MET** | |
| --- | --- | --- | --- | --- | --- | --- | --- | --- | --- | --- | --- | --- | --- |
|  |  | **N (%)** | **95% CI** | **N (%)** | **95% CI** | **N (%)** | **95% CI** | **N (%)** | **95% CI** | **N (%)** | **95% CI** | **N (%)** | **95% CI** |
| Alborz | Female | 247 (67.78%) | (62.77 , 72.41)% | 326 (88.6%) | (84.9 , 91.48)% | 260 (70.46%) | (65.55 , 74.93)% | 345 (93.65%) | (90.6 , 95.76)% | 153 (41.56%) | (36.61 , 46.68)% | 4 (1.1%) | (0.41 , 2.91)% |
|  | Male | 157 (55.77%) | (49.86 , 61.51)% | 238 (78.71%) | (73.72 , 82.97)% | 193 (63.52%) | (57.9 , 68.79)% | 276 (91%) | (87.16 , 93.77)% | 113 (37.62%) | (32.3 , 43.26)% | 7 (2.3%) | (1.1 , 4.77)% |
|  | Total | 404 (62.54%) | (58.72 , 66.22)% | 564 (84.13%) | (81.16 , 86.71)% | 453 (67.32%) | (63.66 , 70.79)% | 621 (92.45%) | (90.16 , 94.24)% | 266 (39.79%) | (36.14 , 43.56)% | 11 (1.64%) | (0.91 , 2.95)% |
| p-value | | 0.002 | | 0.001 | | 0.058 | | 0.201 | | 0.301 | | 0.225 | |
| Ardabil | Female | 187 (57.9%) | (52.38 , 63.23)% | 226 (69.43%) | (64.15 , 74.25)% | 191 (59.09%) | (53.61 , 64.36)% | 300 (92.12%) | (88.63 , 94.6)% | 149 (45.94%) | (40.54 , 51.45)% | 3 (0.88%) | (0.28 , 2.7)% |
|  | Male | 70 (34.26%) | (27.98 , 41.15)% | 117 (47.59%) | (41.37 , 53.89)% | 125 (49.87%) | (43.61 , 56.13)% | 180 (71.8%) | (65.79 , 77.12)% | 128 (51.87%) | (45.6 , 58.07)% | 26 (10.36%) | (7.12 , 14.83)% |
|  | Total | 257 (48.78%) | (44.47 , 53.1)% | 343 (59.94%) | (55.84 , 63.91)% | 316 (55.09%) | (50.95 , 59.15)% | 480 (83.29%) | (79.97 , 86.16)% | 277 (48.52%) | (44.41 , 52.64)% | 29 (4.99%) | (3.48 , 7.11)% |
| p-value | | <0.001 | | <0.001 | | 0.029 | | <0.001 | | 0.163 | | <0.001 | |
| Azerbaijan, East | Female | 277 (58.13%) | (53.62 , 62.51)% | 370 (75.04%) | (71.01 , 78.68)% | 279 (56.36%) | (51.92 , 60.7)% | 425 (86.09%) | (82.71 , 88.89)% | 270 (54.62%) | (50.18 , 58.98)% | 6 (1.22%) | (0.55 , 2.71)% |
|  | Male | 121 (35.8%) | (30.84 , 41.09)% | 227 (52.51%) | (47.77 , 57.21)% | 185 (42.61%) | (38 , 47.35)% | 311 (71.81%) | (67.33 , 75.89)% | 214 (49.69%) | (44.97 , 54.42)% | 33 (7.9%) | (5.66 , 10.94)% |
|  | Total | 398 (48.89%) | (45.45 , 52.34)% | 597 (64.53%) | (61.37 , 67.56)% | 464 (49.94%) | (46.71 , 53.17)% | 736 (79.42%) | (76.67 , 81.93)% | 484 (52.32%) | (49.08 , 55.53)% | 39 (4.34%) | (3.18 , 5.9)% |
| p-value | | <0.001 | | <0.001 | | <0.001 | | <0.001 | | 0.136 | | <0.001 | |
| Azerbaijan, West | Female | 197 (47.88%) | (43.07 , 52.74)% | 249 (58.48%) | (53.7 , 63.09)% | 248 (58.44%) | (53.67 , 63.05)% | 367 (86.27%) | (82.63 , 89.25)% | 219 (51.39%) | (46.62 , 56.13)% | 15 (3.52%) | (2.13 , 5.77)% |
|  | Male | 66 (26.02%) | (20.96 , 31.8)% | 141 (44.05%) | (38.68 , 49.56)% | 135 (42.08%) | (36.77 , 47.59)% | 230 (71.48%) | (66.25 , 76.18)% | 133 (41.54%) | (36.24 , 47.04)% | 36 (11.27%) | (8.23 , 15.25)% |
|  | Total | 263 (39.53%) | (35.87 , 43.32)% | 390 (52.27%) | (48.67 , 55.85)% | 383 (51.4%) | (47.8 , 54.98)% | 597 (79.9%) | (76.86 , 82.64)% | 352 (47.15%) | (43.58 , 50.75)% | 51 (6.85%) | (5.24 , 8.92)% |
| p-value | | <0.001 | | <0.001 | | <0.001 | | <0.001 | | 0.008 | | <0.001 | |
| Bushehr | Female | 349 (59.04%) | (55 , 62.96)% | 471 (77.62%) | (74.1 , 80.78)% | 336 (55.28%) | (51.28 , 59.22)% | 504 (83.19%) | (79.98 , 85.97)% | 307 (50.48%) | (46.49 , 54.47)% | 12 (2.03%) | (1.15 , 3.55)% |
|  | Male | 201 (45.73%) | (41.07 , 50.45)% | 294 (60.63%) | (56.17 , 64.93)% | 246 (50.74%) | (46.26 , 55.22)% | 347 (71.8%) | (67.58 , 75.67)% | 264 (54.75%) | (50.25 , 59.17)% | 30 (6.31%) | (4.44 , 8.91)% |
|  | Total | 550 (53.4%) | (50.32 , 56.45)% | 765 (70.09%) | (67.29 , 72.76)% | 582 (53.27%) | (50.29 , 56.24)% | 851 (78.14%) | (75.57 , 80.51)% | 571 (52.37%) | (49.39 , 55.34)% | 42 (3.93%) | (2.91 , 5.28)% |
| p-value | | <0.001 | | <0.001 | | 0.138 | | <0.001 | | 0.163 | | <0.001 | |
| Chahar Mahaal and Bakhtiari | Female | 193 (42.29%) | (37.81 , 46.89)% | 265 (54.56%) | (50.1 , 58.95)% | 234 (47.98%) | (43.55 , 52.43)% | 404 (82.53%) | (78.86 , 85.68)% | 202 (41.41%) | (37.1 , 45.85)% | 10 (2.04%) | (1.1 , 3.75)% |
|  | Male | 113 (38.32%) | (32.9 , 44.04)% | 194 (50.94%) | (45.9 , 55.96)% | 139 (36.44%) | (31.73 , 41.42)% | 255 (66.96%) | (62.05 , 71.52)% | 158 (41.06%) | (36.22 , 46.07)% | 50 (13.01%) | (9.98 , 16.77)% |
|  | Total | 306 (40.73%) | (37.25 , 44.3)% | 459 (52.97%) | (49.63 , 56.28)% | 373 (42.92%) | (39.65 , 46.25)% | 659 (75.7%) | (72.72 , 78.45)% | 360 (41.25%) | (38.02 , 44.56)% | 60 (6.85%) | (5.35 , 8.73)% |
| p-value | | 0.281 | | 0.29 | | 0.001 | | <0.001 | | 0.917 | | <0.001 | |
| Fars | Female | 349 (58.6%) | (54.59 , 62.5)% | 480 (77.69%) | (74.24 , 80.8)% | 318 (51.52%) | (47.57 , 55.46)% | 535 (86.44%) | (83.5 , 88.93)% | 332 (53.59%) | (49.64 , 57.5)% | 9 (1.46%) | (0.76 , 2.79)% |
|  | Male | 177 (42.28%) | (37.62 , 47.08)% | 296 (57.1%) | (52.79 , 61.3)% | 222 (42.8%) | (38.59 , 47.11)% | 370 (71.26%) | (67.2 , 74.99)% | 250 (48.14%) | (43.85 , 52.46)% | 48 (9.23%) | (7.02 , 12.05)% |
|  | Total | 526 (51.85%) | (48.77 , 54.92)% | 776 (68.29%) | (65.52 , 70.93)% | 540 (47.54%) | (44.64 , 50.46)% | 905 (79.51%) | (77.06 , 81.76)% | 582 (51.11%) | (48.2 , 54.01)% | 57 (5.01%) | (3.88 , 6.44)% |
| p-value | | <0.001 | | <0.001 | | 0.003 | | <0.001 | | 0.067 | | <0.001 | |
| Gilan | Female | 176 (49.31%) | (44.12 , 54.51)% | 242 (65.91%) | (60.92 , 70.57)% | 192 (51.58%) | (46.45 , 56.68)% | 321 (86.51%) | (82.61 , 89.65)% | 186 (49.75%) | (44.64 , 54.87)% | 7 (1.78%) | (0.85 , 3.69)% |
|  | Male | 87 (39.06%) | (32.81 , 45.69)% | 133 (54.57%) | (48.22 , 60.77)% | 109 (44.18%) | (38.02 , 50.53)% | 188 (77.24%) | (71.52 , 82.1)% | 121 (49.49%) | (43.19 , 55.8)% | 18 (7.3%) | (4.63 , 11.33)% |
|  | Total | 263 (45.4%) | (41.36 , 49.5)% | 375 (61.41%) | (57.48 , 65.19)% | 301 (48.65%) | (44.68 , 52.62)% | 509 (82.83%) | (79.63 , 85.63)% | 307 (49.65%) | (45.68 , 53.62)% | 25 (3.97%) | (2.69 , 5.82)% |
| p-value | | 0.017 | | 0.005 | | 0.074 | | 0.003 | | 0.949 | | <0.001 | |
| Golestan | Female | 239 (53.18%) | (48.53 , 57.77)% | 284 (61.4%) | (56.86 , 65.75)% | 226 (48.89%) | (44.34 , 53.47)% | 404 (87.43%) | (84.07 , 90.17)% | 226 (48.41%) | (43.88 , 52.96)% | 6 (1.3%) | (0.58 , 2.88)% |
|  | Male | 132 (41.84%) | (36.49 , 47.39)% | 197 (50.08%) | (45.14 , 55.03)% | 169 (43.02%) | (38.19 , 47.99)% | 293 (74.27%) | (69.7 , 78.37)% | 205 (51.69%) | (46.74 , 56.6)% | 29 (7.38%) | (5.17 , 10.43)% |
|  | Total | 371 (48.49%) | (44.95 , 52.05)% | 481 (56.19%) | (52.84 , 59.49)% | 395 (46.19%) | (42.86 , 49.55)% | 697 (81.37%) | (78.61 , 83.85)% | 431 (49.91%) | (46.57 , 53.26)% | 35 (4.1%) | (2.95 , 5.66)% |
| p-value | | 0.002 | | 0.001 | | 0.087 | | <0.001 | | 0.338 | | <0.001 | |
| Hamadan | Female | 264 (58%) | (53.35 , 62.5)% | 371 (79.96%) | (76 , 83.41)% | 243 (52.48%) | (47.89 , 57.03)% | 409 (88.71%) | (85.49 , 91.28)% | 234 (51.11%) | (46.53 , 55.67)% | 6 (1.28%) | (0.57 , 2.82)% |
|  | Male | 96 (36.76%) | (31.07 , 42.84)% | 160 (47.06%) | (41.77 , 52.43)% | 155 (45.45%) | (40.18 , 50.82)% | 259 (76.13%) | (71.24 , 80.41)% | 172 (51.18%) | (45.82 , 56.5)% | 25 (7.76%) | (5.28 , 11.25)% |
|  | Total | 360 (50.29%) | (46.6 , 53.98)% | 531 (66.04%) | (62.66 , 69.26)% | 398 (49.5%) | (46.03 , 52.98)% | 668 (83.38%) | (80.63 , 85.82)% | 406 (51.14%) | (47.66 , 54.61)% | 31 (4.02%) | (2.84 , 5.67)% |
| p-value | | <0.001 | | <0.001 | | 0.05 | | <0.001 | | 0.985 | | <0.001 | |
| Hormozgan | Female | 169 (55.42%) | (49.54 , 61.16)% | 234 (75.06%) | (69.86 , 79.63)% | 143 (46.76%) | (40.94 , 52.67)% | 280 (89.44%) | (85.46 , 92.43)% | 129 (39.75%) | (34.3 , 45.48)% | 2 (0.67%) | (0.17 , 2.68)% |
|  | Male | 75 (42.15%) | (34.87 , 49.78)% | 126 (55.56%) | (48.83 , 62.09)% | 94 (42.55%) | (36.02 , 49.34)% | 149 (65.8%) | (59.2 , 71.84)% | 115 (49.27%) | (42.62 , 55.95)% | 21 (9.15%) | (6.02 , 13.69)% |
|  | Total | 244 (50.59%) | (45.94 , 55.23)% | 360 (66.86%) | (62.66 , 70.81)% | 237 (44.99%) | (40.62 , 49.44)% | 429 (79.5%) | (75.79 , 82.77)% | 244 (43.76%) | (39.49 , 48.12)% | 23 (4.24%) | (2.83 , 6.32)% |
| p-value | | 0.007 | | <0.001 | | 0.353 | | <0.001 | | 0.033 | | <0.001 | |
| Ilam | Female | 149 (56.05%) | (49.98 , 61.95)% | 193 (68.76%) | (63.07 , 73.94)% | 142 (50.48%) | (44.61 , 56.34)% | 246 (87.65%) | (83.25 , 91.02)% | 139 (49.11%) | (43.26 , 54.99)% | 6 (2.1%) | (0.94 , 4.61)% |
|  | Male | 67 (33.33%) | (27.1 , 40.2)% | 139 (50.78%) | (44.84 , 56.7)% | 117 (42.64%) | (36.87 , 48.61)% | 154 (55.98%) | (50 , 61.79)% | 129 (47.04%) | (41.16 , 53.01)% | 43 (15.87%) | (11.97 , 20.75)% |
|  | Total | 216 (46.28%) | (41.77 , 50.85)% | 332 (59.89%) | (55.73 , 63.9)% | 259 (46.61%) | (42.47 , 50.8)% | 400 (72.01%) | (68.11 , 75.61)% | 268 (48.09%) | (43.94 , 52.27)% | 49 (8.9%) | (6.78 , 11.59)% |
| p-value | | <0.001 | | <0.001 | | 0.065 | | <0.001 | | 0.626 | | <0.001 | |
| Isfahan | Female | 418 (60.23%) | (56.53 , 63.82)% | 510 (71.27%) | (67.83 , 74.48)% | 348 (48.65%) | (44.99 , 52.33)% | 617 (86.26%) | (83.52 , 88.6)% | 351 (48.78%) | (45.13 , 52.45)% | 7 (0.97%) | (0.46 , 2.02)% |
|  | Male | 191 (40.11%) | (35.78 , 44.6)% | 323 (57.82%) | (53.66 , 61.87)% | 226 (40.5%) | (36.48 , 44.64)% | 373 (66.8%) | (62.77 , 70.6)% | 303 (53.93%) | (49.78 , 58.04)% | 39 (6.96%) | (5.12 , 9.4)% |
|  | Total | 609 (52.05%) | (49.18 , 54.91)% | 833 (65.38%) | (62.71 , 67.95)% | 574 (45.08%) | (42.36 , 47.83)% | 990 (77.73%) | (75.35 , 79.94)% | 654 (51.04%) | (48.29 , 53.78)% | 46 (3.6%) | (2.7 , 4.77)% |
| p-value | | <0.001 | | <0.001 | | 0.004 | | <0.001 | | 0.068 | | <0.001 | |
| Kerman | Female | 176 (50.45%) | (45.18 , 55.71)% | 240 (64.69%) | (59.67 , 69.41)% | 181 (48.58%) | (43.49 , 53.69)% | 327 (88.05%) | (84.34 , 90.97)% | 198 (53.15%) | (48.04 , 58.2)% | 2 (0.54%) | (0.13 , 2.15)% |
|  | Male | 91 (34.44%) | (28.93 , 40.41)% | 163 (48.71%) | (43.35 , 54.1)% | 152 (45.27%) | (39.97 , 50.67)% | 225 (67.49%) | (62.26 , 72.33)% | 163 (48.65%) | (43.3 , 54.03)% | 25 (7.56%) | (5.15 , 10.98)% |
|  | Total | 267 (43.53%) | (39.64 , 47.51)% | 403 (57.13%) | (53.43 , 60.76)% | 333 (47.01%) | (43.33 , 50.72)% | 552 (78.33%) | (75.13 , 81.22)% | 361 (51.02%) | (47.32 , 54.71)% | 27 (3.86%) | (2.66 , 5.58)% |
| p-value | | <0.001 | | <0.001 | | 0.381 | | <0.001 | | 0.234 | | <0.001 | |
| Kermanshah | Female | 267 (51.39%) | (47.06 , 55.71)% | 385 (73.75%) | (69.81 , 77.35)% | 286 (54.41%) | (50.09 , 58.66)% | 489 (93.32%) | (90.83 , 95.17)% | 169 (32.44%) | (28.54 , 36.61)% | 6 (1.13%) | (0.51 , 2.49)% |
|  | Male | 135 (37.23%) | (32.38 , 42.36)% | 254 (63.02%) | (58.19 , 67.6)% | 176 (43.33%) | (38.55 , 48.23)% | 335 (82.79%) | (78.79 , 86.18)% | 120 (29.78%) | (25.49 , 34.45)% | 29 (7.09%) | (4.96 , 10.03)% |
|  | Total | 402 (45.55%) | (42.27 , 48.87)% | 639 (69.07%) | (66.02 , 71.97)% | 462 (49.58%) | (46.35 , 52.8)% | 824 (88.73%) | (86.53 , 90.62)% | 289 (31.28%) | (28.36 , 34.36)% | 35 (3.73%) | (2.68 , 5.15)% |
| p-value | | <0.001 | | <0.001 | | 0.001 | | <0.001 | | 0.387 | | <0.001 | |
| Khorasan, North | Female | 222 (56.22%) | (51.25 , 61.06)% | 315 (77.03%) | (72.66 , 80.88)% | 179 (43.62%) | (38.86 , 48.5)% | 364 (89.06%) | (85.6 , 91.77)% | 211 (51.86%) | (46.99 , 56.7)% | 2 (0.48%) | (0.12 , 1.91)% |
|  | Male | 107 (39.38%) | (33.71 , 45.36)% | 177 (50.94%) | (45.64 , 56.21)% | 149 (43.1%) | (37.94 , 48.41)% | 255 (73.42%) | (68.45 , 77.87)% | 193 (55.76%) | (50.45 , 60.94)% | 24 (7.09%) | (4.78 , 10.38)% |
|  | Total | 329 (49.37%) | (45.56 , 53.18)% | 492 (65.08%) | (61.58 , 68.42)% | 328 (43.38%) | (39.87 , 46.96)% | 619 (81.9%) | (78.95 , 84.51)% | 404 (53.65%) | (50.07 , 57.2)% | 26 (3.51%) | (2.39 , 5.11)% |
| p-value | | <0.001 | | <0.001 | | 0.885 | | <0.001 | | 0.286 | | <0.001 | |
| Khorasan, Razavi | Female | 458 (61.93%) | (58.36 , 65.37)% | 608 (80.2%) | (77.19 , 82.89)% | 377 (49.71%) | (46.14 , 53.27)% | 664 (87.72%) | (85.19 , 89.87)% | 426 (56.29%) | (52.73 , 59.79)% | 15 (1.96%) | (1.18 , 3.23)% |
|  | Male | 216 (40.72%) | (36.6 , 44.97)% | 345 (52.67%) | (48.83 , 56.48)% | 294 (44.49%) | (40.71 , 48.32)% | 500 (76.05%) | (72.62 , 79.17)% | 350 (53.4%) | (49.56 , 57.2)% | 33 (4.97%) | (3.55 , 6.91)% |
|  | Total | 674 (53.04%) | (50.29 , 55.78)% | 953 (67.42%) | (64.92 , 69.82)% | 671 (47.28%) | (44.68 , 49.9)% | 1164 (82.3%) | (80.22 , 84.21)% | 776 (54.95%) | (52.34 , 57.53)% | 48 (3.36%) | (2.54 , 4.43)% |
| p-value | | <0.001 | | <0.001 | | 0.05 | | <0.001 | | 0.276 | | 0.002 | |
| Khorasan, South | Female | 186 (49.9%) | (44.83 , 54.97)% | 242 (62.92%) | (57.97 , 67.62)% | 157 (40.67%) | (35.85 , 45.67)% | 343 (88.93%) | (85.38 , 91.7)% | 210 (54.38%) | (49.36 , 59.3)% | 5 (1.31%) | (0.54 , 3.12)% |
|  | Male | 68 (30.35%) | (24.65 , 36.73)% | 104 (37.42%) | (31.9 , 43.28)% | 109 (39.03%) | (33.45 , 44.91)% | 207 (74.25%) | (68.76 , 79.06)% | 142 (50.83%) | (44.95 , 56.7)% | 24 (8.53%) | (5.77 , 12.43)% |
|  | Total | 254 (42.57%) | (38.65 , 46.59)% | 346 (52.22%) | (48.41 , 56.01)% | 266 (39.98%) | (36.31 , 43.77)% | 550 (82.77%) | (79.7 , 85.46)% | 352 (52.89%) | (49.08 , 56.67)% | 29 (4.34%) | (3.03 , 6.18)% |
| p-value | | <0.001 | | <0.001 | | 0.671 | | <0.001 | | 0.367 | | <0.001 | |
| Khuzestan | Female | 338 (63.6%) | (59.35 , 67.64)% | 429 (78.38%) | (74.71 , 81.65)% | 312 (56.7%) | (52.44 , 60.87)% | 483 (88.18%) | (85.18 , 90.64)% | 251 (45.72%) | (41.54 , 49.97)% | 12 (2.18%) | (1.24 , 3.8)% |
|  | Male | 156 (39.52%) | (34.73 , 44.52)% | 275 (61.47%) | (56.83 , 65.91)% | 178 (39.68%) | (35.19 , 44.34)% | 319 (71.35%) | (66.96 , 75.37)% | 209 (46.21%) | (41.58 , 50.9)% | 47 (10.34%) | (7.84 , 13.52)% |
|  | Total | 494 (53.38%) | (50.1 , 56.64)% | 704 (70.76%) | (67.84 , 73.53)% | 490 (49.04%) | (45.9 , 52.18)% | 802 (80.6%) | (78.02 , 82.94)% | 460 (45.94%) | (42.83 , 49.08)% | 59 (5.85%) | (4.56 , 7.49)% |
| p-value | | <0.001 | | <0.001 | | <0.001 | | <0.001 | | 0.879 | | <0.001 | |
| Kohgiluyeh and Boyer-Ahmad | Female | 164 (50.6%) | (45.15 , 56.04)% | 247 (72.32%) | (67.32 , 76.82)% | 143 (41.96%) | (36.8 , 47.29)% | 284 (83.08%) | (78.71 , 86.7)% | 175 (51.29%) | (45.98 , 56.58)% | 5 (1.44%) | (0.6 , 3.43)% |
|  | Male | 62 (26.86%) | (21.51 , 32.99)% | 156 (52.01%) | (46.34 , 57.64)% | 78 (26.06%) | (21.38 , 31.36)% | 138 (45.9%) | (40.32 , 51.59)% | 166 (55.17%) | (49.48 , 60.73)% | 61 (20.16%) | (15.99 , 25.08)% |
|  | Total | 226 (40.74%) | (36.71 , 44.89)% | 403 (62.81%) | (59 , 66.48)% | 221 (34.52%) | (30.92 , 38.29)% | 422 (65.68%) | (61.91 , 69.26)% | 341 (53.11%) | (49.23 , 56.95)% | 66 (10.2%) | (8.09 , 12.79)% |
| p-value | | <0.001 | | <0.001 | | <0.001 | | <0.001 | | 0.327 | | <0.001 | |
| Kurdistan | Female | 178 (45.24%) | (40.35 , 50.21)% | 270 (66.27%) | (61.54 , 70.71)% | 178 (43.74%) | (38.96 , 48.63)% | 376 (92%) | (89 , 94.24)% | 199 (48.9%) | (44.05 , 53.77)% | 4 (0.94%) | (0.35 , 2.49)% |
|  | Male | 90 (33.89%) | (28.4 , 39.85)% | 179 (53.32%) | (47.92 , 58.64)% | 96 (29.04%) | (24.37 , 34.2)% | 267 (79.78%) | (75.11 , 83.76)% | 164 (48.91%) | (43.55 , 54.3)% | 20 (5.91%) | (3.84 , 9.01)% |
|  | Total | 268 (40.71%) | (36.99 , 44.53)% | 449 (60.45%) | (56.87 , 63.92)% | 274 (37.13%) | (33.7 , 40.69)% | 643 (86.51%) | (83.86 , 88.77)% | 363 (48.91%) | (45.31 , 52.52)% | 24 (3.18%) | (2.14 , 4.7)% |
| p-value | | 0.004 | | <0.001 | | <0.001 | | <0.001 | | 0.997 | | <0.001 | |
| Lorestan | Female | 255 (55.1%) | (50.53 , 59.59)% | 345 (71.83%) | (67.62 , 75.68)% | 227 (47.2%) | (42.75 , 51.69)% | 429 (89.29%) | (86.2 , 91.75)% | 293 (60.56%) | (56.09 , 64.86)% | 7 (1.44%) | (0.69 , 3)% |
|  | Male | 96 (35.66%) | (30.13 , 41.61)% | 192 (58.75%) | (53.29 , 64.01)% | 107 (32.7%) | (27.8 , 38.01)% | 233 (71.34%) | (66.15 , 76.03)% | 204 (62.59%) | (57.17 , 67.71)% | 35 (10.64%) | (7.73 , 14.49)% |
|  | Total | 351 (47.99%) | (44.37 , 51.62)% | 537 (66.54%) | (63.2 , 69.73)% | 334 (41.34%) | (37.98 , 44.79)% | 662 (82.04%) | (79.22 , 84.55)% | 497 (61.38%) | (57.95 , 64.7)% | 42 (5.16%) | (3.83 , 6.91)% |
| p-value | | <0.001 | | <0.001 | | <0.001 | | <0.001 | | 0.563 | | <0.001 | |
| Markazi | Female | 195 (55.1%) | (49.85 , 60.24)% | 239 (64.97%) | (59.92 , 69.7)% | 241 (65.58%) | (60.54 , 70.29)% | 331 (90.12%) | (86.59 , 92.8)% | 175 (47.63%) | (42.54 , 52.77)% | 8 (2.22%) | (1.11 , 4.4)% |
|  | Male | 83 (33.21%) | (27.62 , 39.31)% | 158 (49.28%) | (43.82 , 54.76)% | 144 (44.96%) | (39.57 , 50.47)% | 251 (78.16%) | (73.27 , 82.36)% | 148 (46.14%) | (40.72 , 51.64)% | 20 (6.22%) | (4.04 , 9.46)% |
|  | Total | 278 (46%) | (42.05 , 50)% | 397 (57.65%) | (53.91 , 61.3)% | 385 (55.96%) | (52.21 , 59.64)% | 582 (84.54%) | (81.63 , 87.06)% | 323 (46.93%) | (43.22 , 50.68)% | 28 (4.09%) | (2.84 , 5.87)% |
| p-value | | <0.001 | | <0.001 | | <0.001 | | <0.001 | | 0.695 | | 0.009 | |
| Mazandaran | Female | 259 (60.41%) | (55.68 , 64.94)% | 320 (72.7%) | (68.36 , 76.66)% | 236 (53.51%) | (48.82 , 58.14)% | 389 (87.58%) | (84.11 , 90.38)% | 205 (45.64%) | (41.04 , 50.31)% | 3 (0.64%) | (0.21 , 1.99)% |
|  | Male | 140 (44.47%) | (39.02 , 50.05)% | 194 (53.59%) | (48.4 , 58.71)% | 184 (50.98%) | (45.81 , 56.14)% | 277 (76.41%) | (71.72 , 80.54)% | 147 (40.3%) | (35.34 , 45.46)% | 22 (6.25%) | (4.14 , 9.34)% |
|  | Total | 399 (53.69%) | (50.08 , 57.27)% | 514 (64.11%) | (60.72 , 67.37)% | 420 (52.38%) | (48.9 , 55.83)% | 666 (82.56%) | (79.75 , 85.05)% | 352 (43.24%) | (39.85 , 46.7)% | 25 (3.17%) | (2.14 , 4.66)% |
| p-value | | <0.001 | | <0.001 | | 0.477 | | <0.001 | | 0.129 | | <0.001 | |
| Qazvin | Female | 188 (58.49%) | (53 , 63.79)% | 280 (85.8%) | (81.53 , 89.22)% | 155 (47.16%) | (41.76 , 52.63)% | 287 (87.9%) | (83.83 , 91.05)% | 155 (47.76%) | (42.35 , 53.22)% | 1 (0.3%) | (0.04 , 2.1)% |
|  | Male | 84 (37.09%) | (31 , 43.62)% | 164 (59.56%) | (53.61 , 65.25)% | 122 (44.06%) | (38.27 , 50.02)% | 208 (75.64%) | (70.17 , 80.38)% | 151 (54.77%) | (48.8 , 60.6)% | 13 (4.66%) | (2.72 , 7.89)% |
|  | Total | 272 (49.64%) | (45.45 , 53.84)% | 444 (73.8%) | (70.11 , 77.18)% | 277 (45.74%) | (41.78 , 49.76)% | 495 (82.29%) | (79 , 85.16)% | 306 (50.97%) | (46.95 , 54.97)% | 14 (2.29%) | (1.36 , 3.84)% |
| p-value | | <0.001 | | <0.001 | | 0.449 | | <0.001 | | 0.088 | | <0.001 | |
| Qom | Female | 198 (63.84%) | (58.31 , 69.03)% | 258 (80.91%) | (76.16 , 84.9)% | 191 (60.02%) | (54.51 , 65.29)% | 283 (89.03%) | (85.09 , 92.04)% | 178 (56.12%) | (50.59 , 61.51)% | 4 (1.24%) | (0.46 , 3.28)% |
|  | Male | 126 (50.34%) | (44.14 , 56.53)% | 183 (65.83%) | (60.02 , 71.2)% | 171 (61.38%) | (55.48 , 66.96)% | 224 (80.48%) | (75.35 , 84.75)% | 154 (55.57%) | (49.65 , 61.34)% | 19 (6.81%) | (4.37 , 10.45)% |
|  | Total | 324 (57.8%) | (53.66 , 61.84)% | 441 (73.88%) | (70.18 , 77.26)% | 362 (60.65%) | (56.66 , 64.52)% | 507 (85.04%) | (81.93 , 87.7)% | 332 (55.87%) | (51.84 , 59.82)% | 23 (3.84%) | (2.56 , 5.72)% |
| p-value | | 0.001 | | <0.001 | | 0.736 | | 0.004 | | 0.894 | | <0.001 | |
| Semnan | Female | 181 (51.75%) | (46.49 , 56.97)% | 307 (83.84%) | (79.68 , 87.29)% | 167 (45.7%) | (40.63 , 50.87)% | 333 (91.11%) | (87.73 , 93.62)% | 179 (48.98%) | (43.87 , 54.11)% | 2 (0.56%) | (0.14 , 2.22)% |
|  | Male | 100 (36.98%) | (31.39 , 42.94)% | 199 (62.7%) | (57.2 , 67.89)% | 158 (49.47%) | (43.96 , 54.99)% | 255 (80.48%) | (75.72 , 84.5)% | 160 (50.49%) | (44.98 , 55.99)% | 15 (4.87%) | (2.94 , 7.96)% |
|  | Total | 281 (45.32%) | (41.42 , 49.28)% | 506 (74.03%) | (70.59 , 77.2)% | 325 (47.45%) | (43.71 , 51.22)% | 588 (86.18%) | (83.37 , 88.57)% | 339 (49.68%) | (45.93 , 53.43)% | 17 (2.56%) | (1.59 , 4.09)% |
| p-value | | <0.001 | | <0.001 | | 0.327 | | <0.001 | | 0.693 | | <0.001 | |
| Sistan and Baluchistan | Female | 359 (66.23%) | (62.08 , 70.13)% | 409 (74.27%) | (70.41 , 77.79)% | 317 (57.41%) | (53.19 , 61.53)% | 500 (90.71%) | (87.92 , 92.91)% | 288 (51.93%) | (47.71 , 56.11)% | 4 (0.81%) | (0.3 , 2.16)% |
|  | Male | 170 (47.08%) | (41.93 , 52.3)% | 245 (59.19%) | (54.35 , 63.86)% | 187 (45.13%) | (40.34 , 50.01)% | 323 (77.68%) | (73.4 , 81.45)% | 198 (47.52%) | (42.69 , 52.39)% | 33 (7.88%) | (5.64 , 10.9)% |
|  | Total | 529 (58.5%) | (55.22 , 61.7)% | 654 (67.77%) | (64.73 , 70.66)% | 504 (52.12%) | (48.93 , 55.29)% | 823 (85.09%) | (82.68 , 87.22)% | 486 (50.03%) | (46.85 , 53.21)% | 37 (3.86%) | (2.8 , 5.29)% |
| p-value | | <0.001 | | <0.001 | | <0.001 | | <0.001 | | 0.179 | | <0.001 | |
| Tehran | Female | 1035 (59.32%) | (56.98 , 61.62)% | 1388 (78.04%) | (76.05 , 79.91)% | 932 (52.36%) | (50.02 , 54.69)% | 1564 (87.85%) | (86.24 , 89.3)% | 967 (53.77%) | (51.42 , 56.09)% | 19 (1.03%) | (0.66 , 1.6)% |
|  | Male | 660 (51.88%) | (49.12 , 54.64)% | 1004 (71.37%) | (68.94 , 73.69)% | 692 (49.28%) | (46.66 , 51.9)% | 1131 (80.37%) | (78.2 , 82.38)% | 751 (53.18%) | (50.55 , 55.79)% | 62 (4.48%) | (3.5 , 5.72)% |
|  | Total | 1695 (56.19%) | (54.4 , 57.96)% | 2392 (75.1%) | (73.56 , 76.58)% | 1624 (51%) | (49.25 , 52.75)% | 2695 (84.55%) | (83.25 , 85.77)% | 1718 (53.51%) | (51.76 , 55.25)% | 81 (2.55%) | (2.05 , 3.17)% |
| p-value | | <0.001 | | <0.001 | | 0.086 | | <0.001 | | 0.742 | | <0.001 | |
| Yazd | Female | 379 (76.87%) | (72.91 , 80.41)% | 451 (90.56%) | (87.6 , 92.87)% | 336 (67.84%) | (63.58 , 71.82)% | 462 (93.11%) | (90.53 , 95.02)% | 222 (44.92%) | (40.57 , 49.35)% | 1 (0.2%) | (0.03 , 1.38)% |
|  | Male | 221 (48.82%) | (44.21 , 53.45)% | 338 (69.06%) | (64.78 , 73.04)% | 320 (65.57%) | (61.22 , 69.69)% | 381 (78.33%) | (74.45 , 81.77)% | 255 (52.17%) | (47.7 , 56.61)% | 13 (2.6%) | (1.51 , 4.44)% |
|  | Total | 600 (63.45%) | (60.32 , 66.48)% | 789 (79.91%) | (77.26 , 82.31)% | 656 (66.72%) | (63.7 , 69.61)% | 843 (85.79%) | (83.47 , 87.83)% | 477 (48.51%) | (45.38 , 51.65)% | 14 (1.39%) | (0.82 , 2.33)% |
| p-value | | <0.001 | | <0.001 | | 0.452 | | <0.001 | | 0.024 | | 0.001 | |
| Zanjan | Female | 279 (51.76%) | (47.52 , 55.97)% | 404 (74.1%) | (70.25 , 77.61)% | 273 (50.06%) | (45.85 , 54.26)% | 505 (92.51%) | (89.98 , 94.45)% | 298 (54.1%) | (49.9 , 58.25)% | 2 (0.38%) | (0.1 , 1.54)% |
|  | Male | 120 (30.52%) | (26.15 , 35.28)% | 233 (53.13%) | (48.41 , 57.79)% | 195 (44.56%) | (39.95 , 49.28)% | 356 (81.47%) | (77.53 , 84.85)% | 220 (50.09%) | (45.4 , 54.78)% | 23 (5.26%) | (3.52 , 7.81)% |
|  | Total | 399 (42.83%) | (39.67 , 46.04)% | 637 (64.77%) | (61.72 , 67.71)% | 468 (47.61%) | (44.5 , 50.75)% | 861 (87.6%) | (85.39 , 89.53)% | 518 (52.32%) | (49.19 , 55.43)% | 25 (2.55%) | (1.73 , 3.76)% |
| p-value | | <0.001 | | <0.001 | | 0.088 | | <0.001 | | 0.211 | | <0.001 | |
| p-value (between groups and both sexes) | | <0.001 | | <0.001 | | <0.001 | | <0.001 | | <0.001 | | <0.001 | |

Abbreviations: MET: Metabolic equivalent of task; CI: confidence interval.
