# Supplementary material for: Physical activity pattern in Iran: Findings from STEPS 2021
Source: Front Public Health. 2023 Jan 4;10:1036219. doi: 10.3389/fpubh.2022.1036219 (PMC9846211; doi:10.3389/fpubh.2022.1036219)
Supplement: Supplementary Presentation 1 — The map of Iran provinces. [file Presentation_1.PDF]

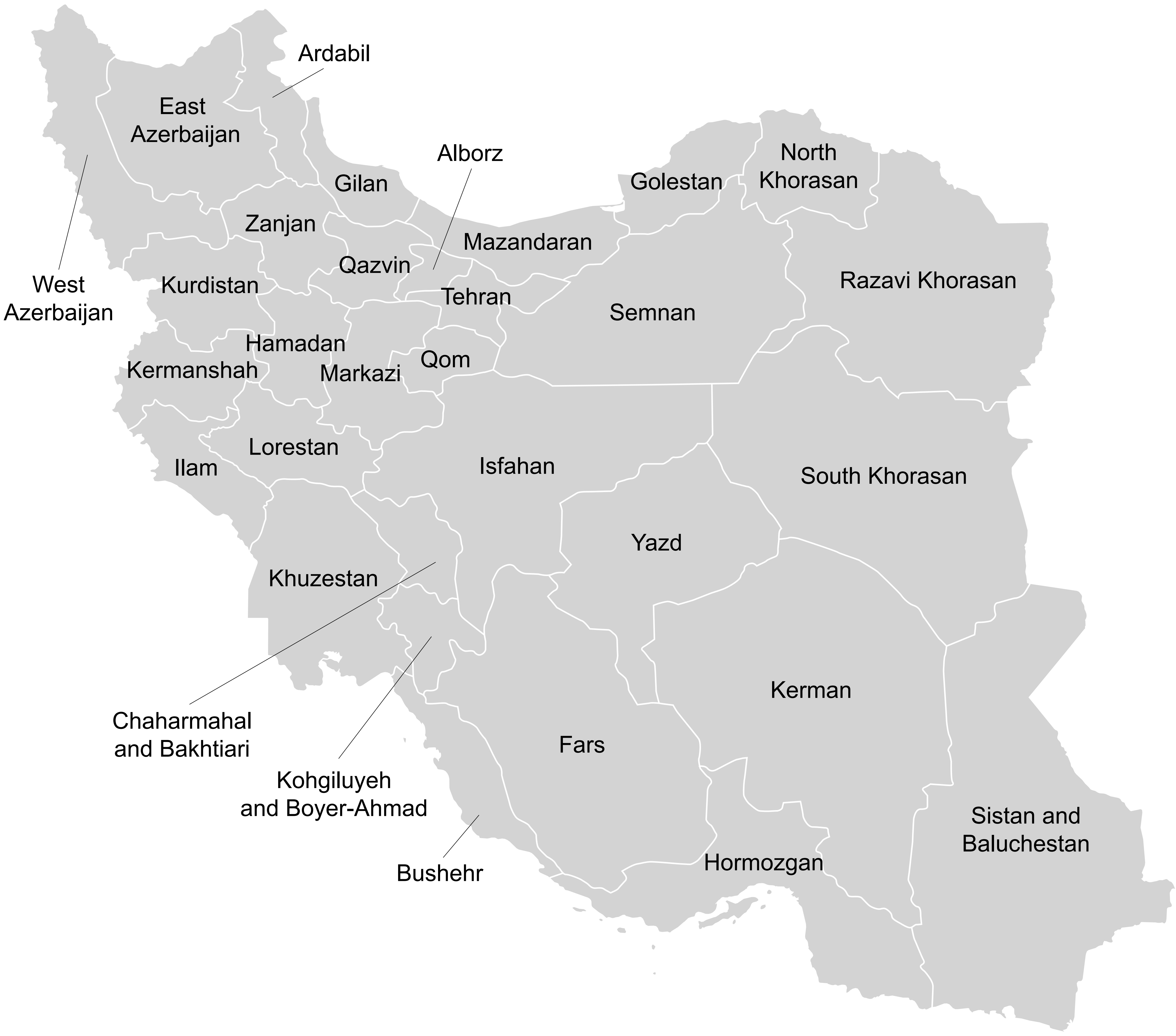
Ardabil
East Azerbaijan
West Azerbaijan
Gilan
Alborz
Golestan
North Khorasan
Zanjan
Mazandaran
Qazvin
Razavi Khorasan
Kurdistan
Tehran
Semnan
Hamadan
Kermanshah
Qom
Markazi
Lorestan
Ilam
Isfahan
South Khorasan
Yazd
Khuzestan
Kerman
Chaharmahal and Bakhtiari
Fars
Kohgiluyeh and Boyer-Ahmad
Sistan and Baluchestan
Hormozgan
Bushehr
